# Upper-layer ozone intrusion promotes wintertime secondary aerosol formation on the ground

**DOI:** 10.1093/nsr/nwaf593

**Published:** 2025-12-27

**Authors:** Yuzheng Wang, Yongchun Liu, Feixue Zheng, Wei Ma, Yusheng Zhang, Chenjie Hua, Xin Chen, Jiali Xie, Zongcheng Wang, Pengkun Ma, Zhiheng Liao, Men Xia, Qi Yuan, Wei Du, Xiaoxi Zhao, Bo Hu, Jiannong Quan, Federico Bianchi, Veli-Matti Kerminen, Tuukka Petäjä, Xiaolei Bao, Shuli Zhao, Jingkun Jiang, Aijun Ding, Markku Kulmala, Douglas R Worsnop

**Affiliations:** Aerosol and Haze Laboratory, Advanced Innovation Center for Soft Matter Science and Engineering, Beijing University of Chemical Technology, Beijing 100029, China; Aerosol and Haze Laboratory, Advanced Innovation Center for Soft Matter Science and Engineering, Beijing University of Chemical Technology, Beijing 100029, China; Aerosol and Haze Laboratory, Advanced Innovation Center for Soft Matter Science and Engineering, Beijing University of Chemical Technology, Beijing 100029, China; Aerosol and Haze Laboratory, Advanced Innovation Center for Soft Matter Science and Engineering, Beijing University of Chemical Technology, Beijing 100029, China; Aerosol and Haze Laboratory, Advanced Innovation Center for Soft Matter Science and Engineering, Beijing University of Chemical Technology, Beijing 100029, China; Aerosol and Haze Laboratory, Advanced Innovation Center for Soft Matter Science and Engineering, Beijing University of Chemical Technology, Beijing 100029, China; Aerosol and Haze Laboratory, Advanced Innovation Center for Soft Matter Science and Engineering, Beijing University of Chemical Technology, Beijing 100029, China; Aerosol and Haze Laboratory, Advanced Innovation Center for Soft Matter Science and Engineering, Beijing University of Chemical Technology, Beijing 100029, China; Aerosol and Haze Laboratory, Advanced Innovation Center for Soft Matter Science and Engineering, Beijing University of Chemical Technology, Beijing 100029, China; Institute of Urban Meteorology, Chinese Meteorological Administration, Beijing 100089, China; Institute of Urban Meteorology, Chinese Meteorological Administration, Beijing 100089, China; Institute for Atmospheric and Earth System Research, Faculty of Science, University of Helsinki, Helsinki 00014, Finland; Nanjing-Helsinki Institute in Atmospheric and Earth System Sciences, Nanjing University, Nanjing 210023, China; Institute for Atmospheric and Earth System Research, Faculty of Science, University of Helsinki, Helsinki 00014, Finland; Institute for Atmospheric and Earth System Research, Faculty of Science, University of Helsinki, Helsinki 00014, Finland; Key Laboratory of Atmospheric Environment and Extreme Meteorology, Institute of Atmospheric Physics, Chinese Academy of Sciences, Beijing 100029, China; Key Laboratory of Atmospheric Environment and Extreme Meteorology, Institute of Atmospheric Physics, Chinese Academy of Sciences, Beijing 100029, China; Institute of Urban Meteorology, Chinese Meteorological Administration, Beijing 100089, China; Institute for Atmospheric and Earth System Research, Faculty of Science, University of Helsinki, Helsinki 00014, Finland; Institute for Atmospheric and Earth System Research, Faculty of Science, University of Helsinki, Helsinki 00014, Finland; Institute for Atmospheric and Earth System Research, Faculty of Science, University of Helsinki, Helsinki 00014, Finland; Hebei Technological Innovation Center for Volatile Organic Compounds Detection and Treatment in Chemical Industry, Hebei Chemical & Pharmaceutical College, Shijiazhuang 050026, China; School of Resources and Environmental Sciences, Bayin Guoleng Vocational and Technical College, Korla 841002, China; State Environmental Protection Key Laboratory of Quality Control in Environmental Monitoring, China National Environmental Monitoring Centre, Beijing 100012, China; State Key Joint Laboratory of Environment Simulation and Pollution Control, School of Environment, Tsinghua University, Beijing 100089, China; School of Atmospheric Sciences, Nanjing University, Nanjing 210023, China; Aerosol and Haze Laboratory, Advanced Innovation Center for Soft Matter Science and Engineering, Beijing University of Chemical Technology, Beijing 100029, China; Institute for Atmospheric and Earth System Research, Faculty of Science, University of Helsinki, Helsinki 00014, Finland; Institute for Atmospheric and Earth System Research, Faculty of Science, University of Helsinki, Helsinki 00014, Finland

**Keywords:** ozone intrusion, secondary aerosol, atmospheric oxidation capacity

## Abstract

Upper-layer ozone (O_3_) intrusion (ULOI) is an important source of surface O_3_, affecting gas pollutants and secondary aerosol formation. However, no robust method has been reported to identify ULOI events based on ground observations and assess their effects on surface atmospheric chemistry. We propose a novel method to identify ULOI events by ranking O_3_ concentrations before dawn and evaluate their contributions to ground-level O_3_ and aerosol formation across China. Our results show that ULOI events occur at a rate of 22%–74% across China, with higher frequency in eastern and southern coastal regions. ULOI enhances ground-level O_3_ by 13–43 ppbv at night and 3–14 ppbv during the day. This increases atmospheric oxidation capacity (AOC) and enhances the contribution of the O_3_ oxidation path to sulfate and secondary organic aerosol (SOA) formation. This study emphasizes the importance of atmospheric layer interactions and the impact of ULOI events on surface atmospheric chemistry.

## Teaser

This study proposed a novel method to identify upper-layer O_3_ intrusion (ULOI) events using ground-based observations. It is found that ULOI occurs frequently and significantly contributes to both nighttime and daytime surface O_3_ levels, boosting atmospheric oxidation capacity and promoting secondary pollution in winter in China.

## INTRODUCTION

Ozone (O_3_) is an important air pollutant on the ground, having adverse effects on human health and the ecosystem. Globally, ∼0.37 million deaths per year have been attributed to O_3_ pollution [1]; a 23% and 33% yield reduction of rice and wheat, respectively, is also associated with surface O_3_ pollution [2]. Surface O_3_ usually shows obvious daily variation with a peak at noon, following the variations of solar radiation. After sunset, O_3_ often decreases continuously due to dry deposition and titration by NO after O_3_ production ceases from the photochemical reactions between nitrogen oxides (NO_x_) and volatile organic compounds. Typically, the O_3_ concentration is around several or even near zero ppbv in the early morning in polluted areas [3]. However, the transport of O_3_ can make anomalously nocturnal ozone enhancement (NOE), which is largely attributed to vertical transport, as rough urban surfaces are not conducive to long-distance horizontal transport of O_3_ at ground level [4].

An upper-layer O_3_ intrusion (ULOI) event, which is usually identified by isotopic measurements and chemical transport models [5,6], represents meteorologically driven O_3_ transport from the upper layer including the residual layer, upper troposphere, and low stratosphere to the ground surface. Special meteorological processes such as sea–land breezes, mountain-valley breezes, low-level jets, tropical cyclones, continental anticyclones, and temperate cyclone systems are usually associated with ULOI events [7–10]. For example, the conversion of sea–land breeze contributes to

the occurrence of coastal low-level jets, which induce turbulent activity, decouple the residual layer and stable layer, and lead to an increase in surface O_3_ concentration [11]. The intrusion of O_3_ from the upper layer often enhances surface O_3_. For instance, the intrusion of stratospheric O_3_ leads to abnormal changes in surface O_3_ on the Tibetan Plateau in summer [12] and increases 15–20 ppbv O_3_ at some stations in the western United States [13]. Ozone is one of the important oxidants in the atmosphere. Its concentration or production rate is taken as a measure of atmospheric oxidation capacity (AOC) [14–16]. Ozone can react with VOCs or inorganic compounds (NO, NO_2_) and contribute to secondary aerosol formation [17,18]. For example, O_3_ can directly react with olefins and terpenoids, contributing to the formation of secondary organic aerosol (SOA) [19]. It also reacts with NO_2_ to produce NO_3_ radicals or N_2_O_5_, driving nocturnal atmospheric chemistry [20]. Ozone also undergoes photolysis, generating OH radicals [21] and promoting secondary aerosol formation [22], and it oxidizes SO_2_ through both multi-phase and homogeneous pathways, leading to the formation of particulate sulfates [23]. Thus, secondary aerosol formation and the subsequent haze pollution in winter, driven by enhanced AOC, have attracted much attention in the atmospheric community [24,25]. ULOI events are typically featured by elevated O_3_ concentrations accompanied by reduced relative humidity (RH) and carbon monoxide (CO) concentrations on the ground [26]. However, the criteria used to identify the ULOI or NOE events are not uniform in the few current studies and might underestimate the ULOI events if a stricter criterion is used. For example, it is attributed to an NOE event if the increment of O_3_ concentration between two adjacent observational data points is >5 ppbv [4]; it is a ULOI event if the observed O_3_ concentration exceeds the 95^th^ percentile of O_3_ concentrations [27]. Such empirical criteria lack general applicability and are often context-specific, making it challenging to extrapolate those findings from localized studies to broader regions or different environments. Although the observational network for surface O_3_ concentration using conventional instruments has been established on regional, national, and even global scales, such as the Global Atmosphere Watch Programme and European Monitoring and Evaluation Programme, a robust and applicable method to identify upper layer O_3_ intrusion based on observed O_3_ concentration changes at the ground to identify ULOI events has not been reported yet. This inhibits the efficient distinguishing of ULOI events as well as evaluating their effects on atmospheric chemistry on different scales, which has not yet been investigated. Here we present a simple method for identifying ULOI events and quantitatively evaluating their impacts on ground-level O_3_ and aerosol chemistry in China. This work highlights the importance of ULOI events in surface atmospheric chemistry and demonstrates the complex interaction between the AOC and aerosol chemistry among different atmospheric layers.

## RESULTS AND DISCUSSION

### ULOI identification

**Figure 1.**
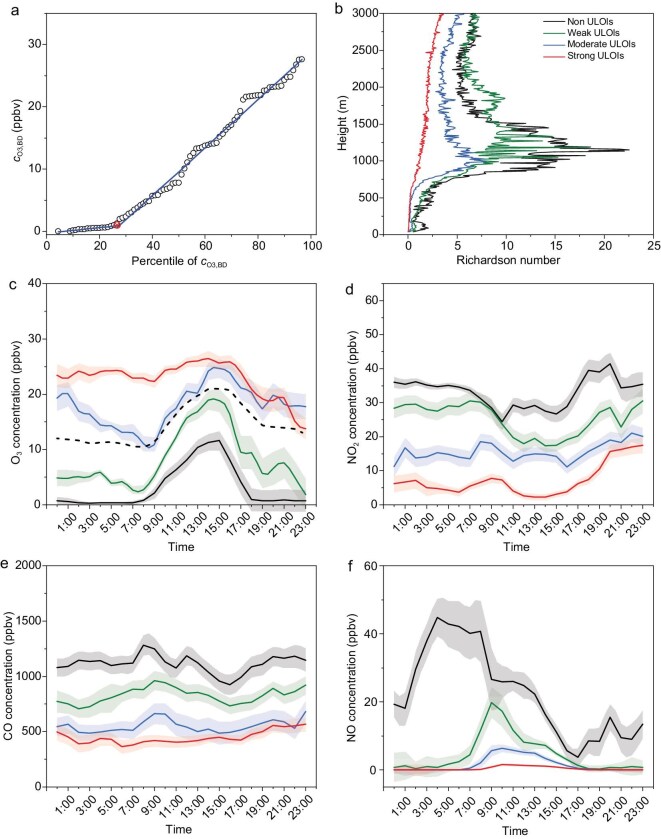
Overview of the distribution of *c*_O3,BD_, averaged bulk Richardson number, and diurnal variations of gas pollutants at BUCT station from 1 December 2020 to 28 February 2021. (a) Scatter plot of *c*_O3,BD_ against its percentiles; red dots indicate the turning point, while blue lines represent linear regressions before and after the turning point. (b) Vertical distribution of the bulk Richardson Number (*R*_i_) with different ULOI intensities [49,50]. (c–f) Diurnal variations of O_3_, NO_2_, CO, and NO with different ULOI intensities. The dashed line in (c) represents the diurnal variation of hourly mean O_3_ during the whole observation. The shaded region around the line represents ±0.2 times the standard deviation.

Ozone at ground level frequently exhibits a clear diurnal variation with a peak at noon and a minimum in the morning [28], influenced jointly by photochemistry production, transport, dry deposition, and chemical reactions (e.g. NO titration). However, in some polluted areas, the diurnal pattern of O_3_ is anomalous, with consistently high concentrations both at night and during the day (Fig. 1c). Upper-layer O_3_ intrusion (ULOI) can lead to anomalously high values of ground-level O_3_ when the O_3_ concentration in the upper layer is higher than that in the boundary layer. This phenomenon is more pronounced at night due to the larger difference in O_3_ concentration between the ground and upper layer than during the day. Hence, the anomalous variation in surface O_3_ concentration can be used to identify the ULOI event (details in Online Methods).

In this work, O₃ concentrations before dawn (*c*_O₃,BD_, 04:00–06:00) are selected to identify ULOI events. This period, while preceding the absolute daily minimum that occurs during rush hours (Fig. 1), offers a more stable baseline that is unaffected by both daytime photochemistry and the sharp rise in anthropogenic emissions, thus more clearly reflecting the O₃ increase from ULOI (Fig. S2). The *c*_O3,BD_ values are selected from the data measured at the BUCT station during the wintertime from 1 December 2020 to 19 February 2021, and their percentile distributions are analyzed. As shown in Fig. 1a, a clear turning point of the O_3_ percentile (*P*_c_) exists in the slopes of the *c*_O3,BD_ versus its percentile, with the *P*_c_ of 25.6%. For the first group, the *c*_O3,BD_ increases slowly (from ∼0 to 1.03 ppbv) in the percentile range of 0%–25.6%, with a slope of 0.044 ppbv/%. This means the absence of ULOI, i.e. non-ULOI (NULOI). In the second group with a percentile of 25.6%–100%, the slope of the *c*_O3,BD_ to percentile increases to 0.39 ppbv/%, which is one order of magnitude higher than that of the first group. Thus, the events in the second group are defined as ULOI events. Similar patterns are observed at other sites in Beijing (Fig. S3), where the *P*_c_ ranges from 25.0% to 38.6%. The variation of the *P*_c_ among different stations might be ascribed to the discrepancy of local micro-environments, including emission sources and topography, that affect the titration rate and deposition rate of O_3_. These results demonstrate that this method is robust for identifying ULOI events. The higher values of the c_O3,BD_ are, the more serious ULOI will be. Therefore, the second group is further divided into three categories with *c*_O3,BD_ percentile of 25.6–50.0 (weak ULOIs), 50.0–75.0 (moderate ULOIs), and 75.0–100.0 (strong ULOIs), respectively. The mean *c*_O3,BD_ are 4.85, 14.67, and 24.73 ppbv, respectively, in the three categories. The number of non-ULOI, weak ULOI, moderate ULOI, and strong ULOI events is 23, 22, 23, and 22, respectively.

To verify the above method for ULOIs identification, the profile of bulk Richardson numbers (*R*_i_) is analyzed. A smaller *R*_i_ means stronger shear forces, indicating an unstable atmosphere that facilitates strong vertical mixing or turbulence, and vice versa [29,30]. As shown in Fig. 1b, the averaged *R*_i_ profile exhibits clear variations at different levels of ULOI intensity, suggesting a potential link between turbulence conditions and O_3_ concentration enhancements. The non-ULOIs have the largest *R*_i_ at different altitudes from the ground to 3000 m, suggesting the most stable atmosphere among the four categories. Comparatively, the lowest *R*_i_ appears in strong ULOIs, with the *R*_i_ in weak and moderate ULOIs being between the non-ULOIs and strong ULOIs. It should be noted that peaks of *R*_i_ profiles that appear in the low layer of the troposphere (700–1500 m) can suppress the vertical transport of O_3_ and other pollutants between the boundary layer and the free troposphere, in particular, during non-ULOI events. Similar vertical profiles of *R*_i_ were also observed at 08:00 and 20:00 (Fig. S4). The air masses of different ULOI events mostly come from heights below 800–850 hPa, which is an altitude of ∼2000–1500 m (Fig. S5). This altitude belongs to the free troposphere or residual layer in winter in Beijing. The peak values of *R*_i_ below 1500 m decrease notably with the increase in *c*_O3,BD_, which means that the stability of the boundary layer is closely related to the strength of the ULOI events (Fig. S6). The O_3_ flux under different intensities of ULOI (Fig. S7) further consolidates the identification of ULOI events according to the *c*_O3,BD_. Briefly, the weakest O_3_ flux appears in non-ULOIs, while the strongest O_3_ flux appears in strong ULOIs. As shown in Fig. 1c–f, the diurnal variations of O_3_ and other gaseous pollutants (e.g. NO, NO_2_, and CO) in these four cases further confirm the validity of the ranking method of *c*_O3,BD_ in reflecting ULOI intensity. This is further verified by higher concentrations of O_3_ in ULOI events than in non-ULOI events, accompanied by reverse relationships of other pollutants across typical stations in China (Fig. S8). For strong ULOI events, O_3_ in the upper layers is transported downwards, accompanied by dilution of NO, NO_2_, and CO at the ground, resulting in the highest O_3_ and the lowest other three pollutants due to an unstable atmosphere facilitating the vertical exchange of pollutants. On the contrary, a stabilized atmosphere in non-ULOI events suppresses the vertical exchange of pollutants, resulting in the lowest O_3_ and the highest other three pollutants. These results further confirm that the values of *c*_O3,BD_ strongly depend on the vertical transport of air mass and can be reliably used to identify ULOI events.

### Prevalence of upper-layer O_3_ intrusion and its impacts

**Figure 2.**
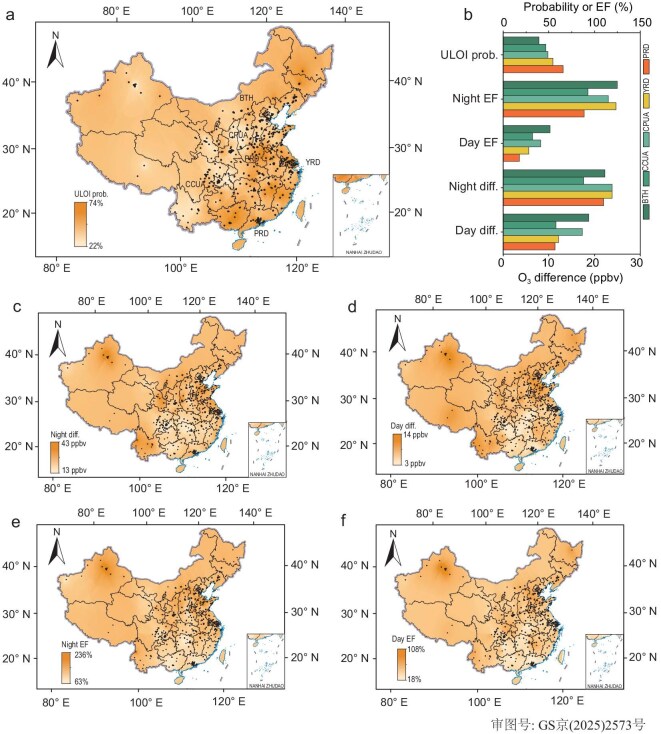
The spatial distribution of ULOI probability, the difference in O_3_ concentrations between ULOI and NULOI, and the O_3_ enhancement factor (EF) across China from December 2020 to February 2021. (a) The spatial distribution of ULOI probability. (b) The ULOI probability, O_3_ difference, and EF during nighttime and daytime at the main urban agglomerations in China. (c and d) The difference in O_3_ concentrations between ULOI and NULOI during nighttime and daytime. (e and f) The EFs of O_3_ during nighttime and daytime. The black dots in the figures represent the coordinates of the observed sites used here. Beijing-Tianjin-Hebei Urban Agglomeration (BTH), Central Plains Urban Agglomeration (CPUA), Yangtze River Delta Urban Agglomeration (YRD), Pearl River Delta Urban Agglomeration (PRD), and Chengdu-Chongqing Urban Agglomeration (CCUA) are the main urban agglomerations in China.

As discussed above, after excluding the effect of horizontal transport, the *P*_c_ marks the demarcation between non-ULOI events and ULOI events. Thus, the probability of ULOI events (*P*_ULOI_) can be defined by *P*_ULOI_ = 1–*P*_c_. The *P*_ULOI_ values across China (Fig. 2a) are calculated using observed O_3_ concentrations obtained from the China National Environment Monitoring Center (https://www.cnemc.cn/) from December 2020 to February 2021. Overall, ULOI events are prone to occur in the eastern and southern regions of China. Especially, the probability of ULOI events is as high as ∼70% in the coastal regions. The development of coastal low-level jets during sea–land breeze circulation shifts may disrupt the coupling between the residual layer and the stable boundary layer, thereby facilitating the onset of ULOI [11]. The probability of ULOI in the Yangtze River Delta Urban Agglomeration (YRD) and the Pearl River Delta Urban Agglomeration (PRD) is ∼54.4% and 65.5% respectively. In central China, such as the Beijing-Tianjin-Hebei Urban Agglomeration, the probability of ULOI is relatively low (∼38.9%). The probability of ULOI means that ground-level O_3_ tends to be affected frequently by vertical transport in winter. The spatial distribution of ULOI probability may be related to various factors, such as altitude, terrain, meteorological processes, etc., which require further investigation in the future. It is noteworthy that regions such as eastern China, which have a high probability of ULOI, are also economically developed areas facing severe O_3_ pollution. Thus, the high probability of ULOI may exacerbate O_3_ pollution in these regions.

Figure 2c and d illustrates the O_3_ concentration difference between ULOI and non-ULOI. ULOI events increased ground-level O_3_ concentrations by 13–43 ppbv on average during the nighttime and 3–14 ppbv during the daytime, with the maximal increase observed in the eastern coast regions of China. In the vast southern regions of China, the increments of nighttime ground-level O_3_ concentrations are relatively low, ∼13 ppbv, although the P_ULOI_ values are as high as 70%. The net contribution of ULOI to ground-level O_3_ concentrations can be expressed in terms of the enhancement factor (*EF*),


(1) \begin{equation*}EF = \frac{{{c}_{{\rm O}3,{\rm ULOI}} - {c}_{{\rm O}3,{\rm NULOI}}}}{{{c}_{{\rm O}3,{\rm NULOI}}}} \times 100\% ,
\end{equation*}

where *c*_O3__,__ULOI_ is the mean O_3_ concentration of ULOI events, and *c*_O3__,__NULOI_ represents the mean O_3_ concentration of non-ULOI events. ULOI events lead to nighttime *EF*s ranging from 63% to 236% nationwide (Fig. 2e) and daytime *EF*s varying from 18% to 108% (Fig. 2f). These results suggest that ULOI events could have significant impacts on atmospheric chemical processes as discussed earlier. There are five major urban agglomerations in China, which are the Beijing-Tianjin-Hebei Urban Agglomeration (BTH), Central Plains Urban Agglomeration (CPUA), Yangtze River Delta Urban Agglomeration (YRD), Pearl River Delta Urban Agglomeration (PRD), and Chengdu-Chongqing Urban Agglomeration (CCUA). Compared to the non-ULOI period, ULOI events resulted in nighttime (daytime) O_3_ elevations of 22.3 (18.7), 23.9 (17.3), 23.8 (12.1), 22.0 (11.38), and 17.6 (11.6) ppbv, respectively, in those five urban agglomerations. This has a significant adverse effect on O_3_ control and may broadly influence secondary aerosol production.

### Impact of ULOIs on aerosol chemistry

As shown in Fig. 1c, O_3_ increases significantly in ULOIs, with higher enhancement in nighttime (18:00–06:00) than in daytime (07:00–17:00). The mean daytime (nighttime) O_3_ concentration increases from 8.31 (3.53) to 25.40 (20.80) ppbv from non-ULOIs to strong ULOIs. Such O_3_ enhancements in ULOIs will affect AOC and aerosol chemistry. During our observations, the mass concentrations of both PM_2.5_ and its components monotonically decrease as a function of ULOI intensity (Table S1), due to enhanced atmospheric diffusion conditions (Fig. 1b). This is similar to the dilution of anthropogenic gases (e.g. SO_2_, NOx, CO) as shown in Fig. 1e and f. Besides mass concentration, the aerosol composition shows a clear difference between non-ULOIs and ULOIs. The mass fractions of sulfate (SO_4_^2−^) and OA increase as a function of ULOI (Fig. S9). For SO_4_^2−^, its mass fractions are 12%, 13%, 16%, and 18% in non-ULOI, weak ULOI, moderate ULOI, and strong ULOI, respectively, and the corresponding mass fractions of OA are 29%, 28%, 29%, and 36%. This means that the ULOI events change the composition of PM_2.5_, which might result from different aerosol compositions in the vertical direction and/or changes in the formation rate at the surface caused by variations in the concentration of oxidants and precursors [31–33].

**Figure 3.**
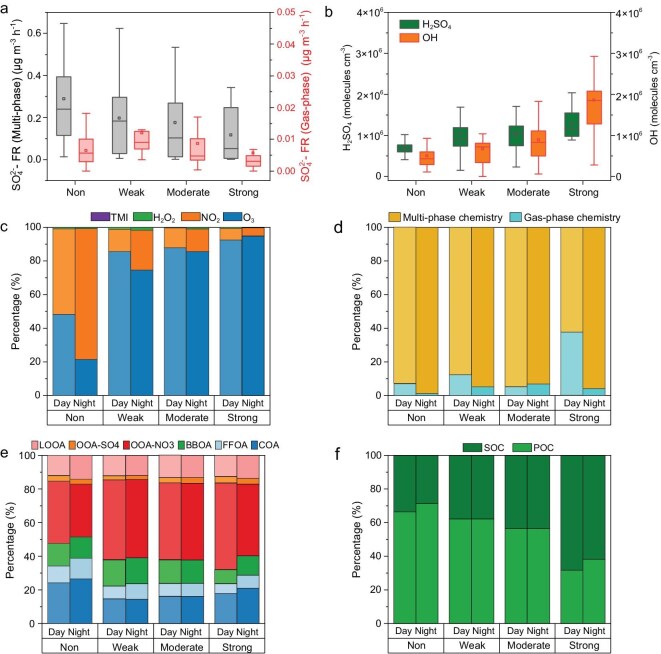
Overview of the dependence of sulfate formation and OA sources on the intensities of ULOI at BUCT station. (a) Sulfate formation rates of multi-phase and gas-phase chemistry with different intensities of ULOI. (b) The concentrations of H_2_SO_4_ and OH radicals during daytime with different intensities of ULOI. (c) Fraction of sulfate formation rates via different formation pathways in a liquid aerosol. (d) Contribution of multi-phase and gas-phase chemistry to the formation rates of sulfate with different intensities of ULOI. (e) Contribution of different OA factors to OA in different intensities of ULOI. (f) Contribution of POC and SOC to TOC in different intensities of ULOI. All data used in (c–f) are median values in different intensities of ULOI.

Secondary sulfate on the ground level mainly forms through two pathways, i.e. multi-phase chemistry and gas-phase chemistry. In the former case, dissolved SO_2_ (or S(IV)) in aerosols is oxidized by oxidants including H_2_O_2_, O_3_, and NO_2_, and catalytically oxidized by transition metal ions (TMIs), whereas the latter involves oxidation of gas-phase SO_2_ by OH radicals to produce H_2_SO_4_ followed by condensation onto aerosol seeds [34–36]. Figure 3a shows the formation rates of sulfate via multi-phase chemistry and gas-phase chemistry, in which multi-phase chemistry is the dominant sulfate gas-phase pathway. As the ULOI intensity increases, however, the sulfate formation rates, limited by both precursors, relative humidity, liquid water content (LWC), etc., gradually decrease in both pathways.

As for multi-phase chemistry, oxidation of S(IV) by NO_2_ and O_3_ are the dominant oxidation pathways, whereas the oxidation paths related to TMIs and H_2_O_2_ were unimportant during our observations (Fig. 3c). In addition, the contribution of the O_3_ oxidation pathway during daytime (nighttime) increases from 48.3% (21.5%) to 92.3% (94.8%), and the NO_2_ oxidation pathway decreases from 50.8% (77.8%) to 7.0% (4.9%) from non-ULOIs to strong ULOIs. This is consistent with the trends of O_3_ and NO_2_ concentrations as a function of the ULOI intensity (Fig. 3). Since the four major aqueous-phase oxidation pathways occur in aerosol liquid water, relative humidity (RH) and aerosol liquid water content primarily influence sulfate formation by modulating the volume of the reaction medium and the concentration of dissolved S(IV), thereby exerting a comparable effect across these pathways in a conceptual sense. However, the variation in aerosol pH has significantly different impacts on different pathways [23,32]. A slight decline in pH, from 5.24 ± 0.48 to 3.95 ± 0.80 on average, was found from non-ULOIs to strong ULOIs as shown in Fig. S10. This means the increased relative contribution of O_3_ oxidation to sulfate formation in strong ULOI events could be explained by enhanced O_3_ concentrations, even if it is offset by decreased aerosol pH (Fig. S11). The decreased relative contribution of NO_2_ oxidation to sulfate formation, however, could be explained by the harmonious decrease in NO_2_ concentration and aerosol pH. The phenomenon that the contribution of the O_3_ oxidation pathway to sulfate formation increases with the enhancement of ULOI was further confirmed by the WRF-Chem model (Figs S12 and S13), highlighting the possible impacts of ULOI events on aerosol chemistry on ground surfaces. Compared to the multiphase oxidation of S(IV), the contribution of heterogeneous condensation from H_2_SO_4_ vapor increases as the ULOI intensity increases, as shown in Fig. 3d. The upper layer is relatively clean as far as aerosol concentration is concerned, thus the condensation sink decreases with an increase in the ULOI intensity (Fig. S14). However, H_2_SO_4_ vapor, from the reaction between SO_2_ and OH radicals, increases with a rise in the ULOI intensity (Fig. 3b). Despite the lower SO_2_ concentrations in strong ULOI events, the enhanced OH concentrations with ULOI intensity could explain the increase of H_2_SO_4_ concentrations, as illustrated by the change in average OH concentrations (Fig. 3b) calculated using the parametric method [37–40]. In summary, ULOIs can modify the contribution of the different pathways to sulfate formation by changing precursor concentrations, aerosol pH, and OH radical concentrations.

In addition to SO_4_^2−^, the percentage of OA increases at the elevated intensity of ULOIs. As shown in Fig. 3e, the proportion of primary organic aerosols (POAs, including COA, FFOA, and BBOA) gradually decreases during daytime (nighttime) from 47.6% (51.4%) to 32.0% (40.1%), while the fraction of SOA (including LOOA, OOA-SO_4_^2−^, and OOA-NO_3_^−^) gradually increases from 52.4% (48.6%) to 68.0% (59.9%), with the most obvious increase being mainly in the fraction of OOA-NO_3_^−^ as the intensity of ULOIs rises. For example, the fractions of OOA-NO_3_ in weak and moderate ULOI events are almost constant at daytime (∼46%). They are higher than that in weak ULOI events (37.1%) but lower than that in strong ULOI events (51.6%). The elevated O_3_ concentrations in strong ULOIs may accelerate the formation of NO_3_ radicals [41–43] and hence elevate the contribution of OOA-NO_3_^−^. However, the decreased NO_2_ and PM_2.5_ concentrations and low RH are not favorable for the formation and hydrolysis of N_2_O_5_. Thus, the proportion of NO_3_^−^ through N_2_O_5_ hydrolysis may be inhibited. The contribution of secondary organic carbon (SOC), calculated utilizing the minimum R-squared method proposed by Millet *et al.* [44] and Wu *et al.* [45], to total organic carbon (TOC) also elevates on average by 20% in daytime and 16% at nighttime from non-ULOI to ULOI, respectively (Fig. 3f). The elevated proportion of SOA and SOC could be explained by the vertical transport of upper air or the elevated oxidation capacity, while more detailed measurements and modeling are needed to explain these results in the future. In summary, we can conclude that from a perspective of chemical kinetics, ULOI events facilitate the formation of secondary aerosols including sulfate and SOA due to an increase in O_3_ concentration although the concentration of PM_2.5_ on ground surfaces is determined by the dilution effect of PM_2.5_ and its precursors.

**Figure 4.**
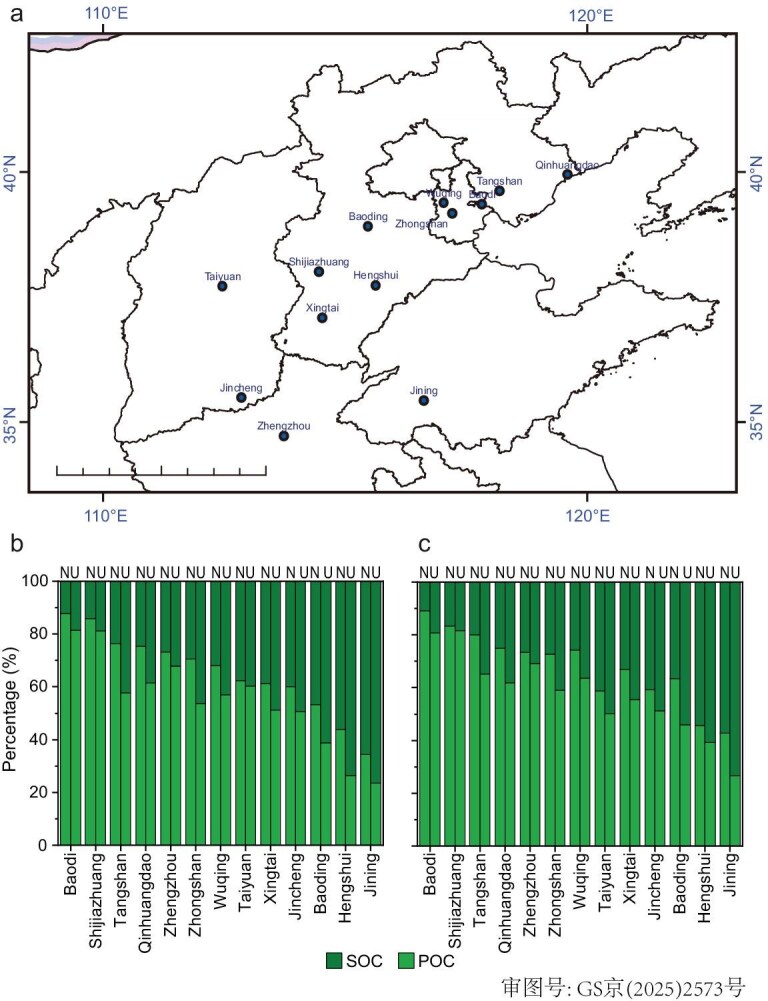
Overview of OC composition as a function of ULOI. (a) The location of stations used in this study. (b and c) The POC and SOC percentage during non-ULOI and ULOI events during daytime and nighttime. The N and U represent non-ULOI and ULOI events.

Besides the BUCT station, we selected 13 stations, on which OC and EC data are available, in the North China Plain to confirm the significant impact of ULOI events on ground-level organic aerosol formation. As shown in Fig. 4, the proportion of SOC varies across the 13 sites. During the daytime, the fractions of SOC range from 12% to 76%, while they range from 11% to 73% at night. In the ULOI events, the fractions of SOC exhibit significant increases (*P* < 0.05) both in the daytime and nighttime compared to those in non-ULOI events. Specifically, the SOC proportions increase by 2%–19% during the daytime and by 4%–18% at night, with average increases of 10% and 11%, respectively. The enhancements of the SOC fraction due to ULOI are close to those observed in Beijing. This indicates that the influence of ULOI on the composition of OC is widespread. In conclusion, our results demonstrate that ULOI events definitely alter ground-level O_3_ concentrations, sulfate, and SOA formation. These findings highlight the need for greater attention to the impacts of ULOI events on ground atmospheric chemistry. Evaluating these effects is crucial for understanding their broader implications and guiding effective atmospheric management strategies. Currently, observational data of atmospheric pollutants including O_3_ are easily accessed based on plenty of ground-based observation networks in the world. The method developed based on observations in this study provides a new avenue to reveal the complex interaction between the AOC and aerosol chemistry among different atmospheric layers, while more quantitative comparisons are required in the future with other methods, such as chemical transport models. Additionally, severe O_3_ pollution usually occurs in summer in Beijing, and thus, it should be meaningful to identify ULOI events and reveal their impacts on summertime O_3_ pollution in the future. More comprehensive analyses, including the synoptic evolution and meteorological conditions, would further validate the impact of ULOI on O_3_ and related pollutants.

## MATERIALS AND METHODS

According to the law of conservation of mass, O_3_ concentration at a given time (${c}_{{O}_3{,}{t}_i}$) is,


(2) \begin{eqnarray*}
{c}_{{\rm O}_3{,}{t}_i} &=& {c}_{{\rm O}_3{,}{t}_{i - 1}} + \mathop \int \nolimits_{{t}_{i - 1}}^{{t}_i} {T}_{{\rm O}_3}dt + \mathop \int \nolimits_{{t}_{i - 1}}^{{t}_i} {P}_{{\rm O}_3}dt\nonumber\\
&&- \mathop \int \nolimits_{{t}_{i - 1}}^{{t}_i} {d}_{{\rm O}_3}dt - \mathop \int \nolimits_{{t}_{i - 1}}^{{t}_i} {R}_{{\rm O}_3}dt,
\end{eqnarray*}

where ${c}_{{\rm O}_3{,}{t}_{i - 1}}$ is the concentration of O_3_ at the time of *t*_*i*−1_; ${T}_{{\rm O}_3}$, ${P}_{{\rm O}_3}$, ${d}_{{\rm O}_3}$, and *R*_O_3__ are the net transport rate, production rate, dry deposition rate, and reaction rate of O_3_ with other species such as NO, respectively. In urban environments, the production rate of O_3_ is zero at night. Thus, O_3_ is continuously titrated by NO and dry deposition after sunset so that O_3_ concentrations should decrease to a regional background value (near zero) before dawn (04:00–06:00) of the next day if the net transport does not occur. The transport or nocturnal ozone enhancement can come from either the vertical or horizontal direction, which makes the O_3_ concentration before dawn higher than on days without transport. If all the O_3_ concentrations before dawn (*c*_O3,BD_) are ranked according to their percentiles during the observations, a higher slope should be expected than the counterpart (Fig. 1c) when vertical transport is active due to the net contribution from the vertical direction to ground-level after excluding the days with horizontal transport. Therefore, the turning point of the *c*_O3,BD_ versus its percentiles should be the critical value (*c*_O3,c_) for ULOI events at an observation site. The turning point is obtained by fitting a piecewise linear function using nonlinear least squares. ULOI events occur if the ratio of the *c*_O3,BD_ to *c*_O3,c_ (*R*_O3, BD _=* c*_O3,BD_/*c*_O3,c_) is >1.0 after excluding horizontal transport. Thus, the O_3_ ranking method is for the first time proposed to characterize ULOI events in an objective, quantifiable, and replicable manner.

It should be noted that the O_3_ concentration on the ground is greatly affected by photochemistry during daytime. Thus, the method applied in the morning is unsuitable for identifying ULOI events during the daytime. However, the outcome of ULOI events is leading to an increase in ground-level O_3_ concentrations along with decreases in NO, NO_2_, and CO concentrations as shown in Fig. 1. The changes in chemical composition in the early morning during ULOI events may affect atmospheric chemistry, including photochemistry and direct oxidation reactions, as discussed above. This is consistent with previous findings that nocturnal ozone enhancement (NOE) and the LLJ events observed at night promote ozone pollution on the ground the following day [4,46,47].

And it also should be noted that the *P*_c_ value reflects the proportion of non-ULOI events to the total number of days. This may vary with the size of the dataset or the duration of observations (Fig. S15) because ULOI events are driven by meteorological conditions. The *P*_c_ value was derived using the whole dataset, indicating a specific value during our observations. But in reality, this does not affect the ability of this method to distinguish ULOI events from non-ULOI events (Fig. S15). Although the concentrations of NO and other pollutants may decrease on windy days due to the dilution effect, the titration rate of O_3_ by NO remained stable at different percentiles of *c*_O3,BD_ throughout the observation period (Fig. S16). This means that the titration effect may have little influence on the identification of ULOI events.

To exclude those days with horizontal transport, horizontal transport was identified using the method proposed by An [48]. Briefly, this transport is dominated by vertical transport at night, whose ranking value is larger than the turning point if CO concentration decreases along with friction velocity and boundary layer height increase; otherwise, if CO concentration increases, accompanied by decreased or unchanged friction velocity and planetary boundary layer height, the transport event is dominated by horizontal transport. According to this criterion, no horizontal transport event was identified during the study period at the BUCT station. Horizontal transport events were also excluded in other stations across China using the same method. The identified ULOI events were further checked by the variations of CO, NO, and wind speed (WS) before dawn, classified according to the ranking percentiles of *c*_O3,BD_.

Detailed descriptions of the field observations, reanalysis of data, calculation of aerosol AWC and pH, and other materials and methods can be found in the supporting information.

## Supplementary Material

nwaf593_Supplemental_File

## Data Availability

All data are available in the main paper and the Supplementary Data.
