# Supplementary material for: Upper-layer ozone intrusion promotes wintertime secondary aerosol formation on the ground
Source: Natl Sci Rev. 2025 Dec 27;13(2):nwaf593. doi: 10.1093/nsr/nwaf593 (PMC12831027; doi:10.1093/nsr/nwaf593)
Supplement: nwaf593_Supplemental_File [file nwaf593_supplemental_file.docx]

Supplementary Materials for

**Upper-layer ozone intrusion promotes wintertime secondary aerosol formation on the ground**

Yuzheng Wang *et al.*

*Corresponding author. Email: liuyc@buct.edu.cn (Yongchun Liu), jnquan@ium.cn (Jiannong Quan), and Douglas R. Worsnop (worsnop@aerodyne.com)

**This file includes:**

Text 1 to Text 8

Tables S1 to S2

Figs. S1 to S18

Text 1: Field observations

Field observations were carried out at Aerosol and Haze Laboratory, Beijing University of Chemical Technology (AHL/BUCT station, Lat. 39°56ʹ31ʺand Lon. 116°17ʹ52ʺ) from December 1, 2020, to February 28, 2021. This station is located on the rooftop of the main teaching building, near residential areas and traffic arteries, and is a typical urban observation station. The details have been described in previous works [1-3].

The instruments used in this field observation are presented in Table S2. Briefly, the mass concentration of PM_2.5_ was measured by a Taper Element Oscillating Microbalance (TEOM 1405-DF, Thermo Fisher). Water-soluble ions (Na^+^, K^+^, Ca^2+^, Mg^2+^, Cl^-^, NO_3_^-^, and SO_4_^2-^) of PM_2.5_ and gas pollutants (HCl, HNO_3_, SO_2_, and NH_3_) were measured by an analyzer for Monitoring AeRosols and Gases in Ambient Air (MARGA 2060R, Metronhm Process Analytics). The non-refractory PM_2.5_ compositions (nitrate, sulfate, chloride, organic, and ammonium) were measured using a Time-of-Flight Aerosol Chemical Speciation Monitor (ToF-ACSM, Aerodyne). Trace gases, including NO_x_, SO_2_, CO, and O_3_, were measured using the corresponding analyzer (Thermo Scientific, 42i, 43i, 48i, 49i). Meteorological parameters, including pressure, relative humidity, temperature, wind speed, and direction, were measured using weather stations (AWS310, Vaisala).

The MARGA was externally calibrated monthly and internally calibrated hourly. The detection limits of Cl^-^, NO_3_^-^, SO_4_^2-^, Na^+^, NH_4_^+^, K^+^, Mg^2+^, and Ca^2+^ were 0.01, 0.05, 0.04, 0.05, 0.06, 0.09, 0.06 μg m^-3^. The ACSM was calibrated every three months using NH_4_NO_3_, NH_4_NO_3,_ and NH_4_Cl solutions. Trace-gas measurement instruments were calibrated every week with the corresponding standard gases. The detection limits of NO_x_, SO_2_, CO, and O_3_ were 0.05, 0.05, 40.00, and 0.50 ppbv respectively. To validate the influence of vertical transport of O_3_ to aerosol chemistry, OC and EC concentrations observed in 12 stations from the China National Environment Monitoring Center (<https://www.cnemc.cn/>) were also used in this study.

The observed O_3_ concentrations obtained from the China National Environment Monitoring Center (<https://www.cnemc.cn/>) were used to calculat the possibility of ULOI events across China. The limited number of stations in the western region brings a certain degree of uncertainty, so we mainly focus on the eastern and southern regions of China when discussing the impacts of ULOI events on secondary aeorol formation and ground surface O_3_ concentrations.

Text 2: Reanalysis data

The Copernicus Atmosphere Monitoring Service (CAMS) reanalysis data [4], including the u-component of wind, v-component of wind, vertical velocity, and O_3_ concentrations, were acquired from Copernicus (<https://www.copernicus.eu>). The reanalysis data were used to characterize the vertical distribution of O_3_ concentration, horizontal wind speed, and vertical wind speed. The time resolution of CAMS reanalysis data is 3 hours. The vertical resolution of reanalysis data is pressure levels: 1000, 950, 925, 900, 850, 800, 700, 600, 500, 400, 300, 250, 200, 150, 100, 70, 50, 30, 20, 10, 7, 5, 3, 2, 1 hPa. In this study, reanalysis data from 1000 hPa to 600 hPa were used.

The fifth generation European Centre for Medium-Range Weather Forecasts (ECMWF) reanalysis data (ERA5) from Copernicus, including boundary height and friction velocity, was used to distinguish whether the transport source is vertical or horizontal. The time resolution of ERA5 data is 1 hour.

Text 3: Calculation of aerosol AWC and pH

The aerosol water content (AWC) and pH were calculated using the thermodynamic equilibrium model, ISORROPIA Ⅱ [5], with an assumption that the aerosol system was in equilibrium. In this study, the model was fed with the variables, including temperature, relative humidity, and the total concentration of NH_3_, H_2_SO_4_, Na, HCl, and HNO_3_. The model setting is in “forward” mode and in the “metastable” phase state. The pH was calculated according to the following formula:

$pH=-{log}_{10}\frac{1000\gamma_{H^{+}}c_{H^{+}}}{AWC}$ (2)

where γ_H+_ is the activity coefficient of H^+^ (assumed to be 1.0) [1, 5, 6]. The dataset was selected when RH>30% to reduce the uncertainty of AWC calculations.

Text 4: Calculation of bulk Richardson number

Bulk Richardson numbers (*R*_i_) is a dimensionless parameter used in meteorology to assess atmospheric stability and vertical mixing or turbulence. The *R*_i_ is calculated according to equation (3), to confirm the ULOIs.

$R_{i}\left( z \right)=\frac{g\left( z-z_{0} \right)}{\theta\left( z \right)}\frac{\left[ \theta\left( z \right)-\theta\left( z_{0} \right) \right]}{\left[ {u\left( z \right)}^{2}+{v\left( z \right)}^{2} \right]}$ (3)

Where *g* is the acceleration due to gravity (9.8 m s^-2^), z_0_ is the height above sea level, *θ* is the potential temperature, *u* and *v* are the zonal and meridian wind components, respectively [7, 8]. The radio-sounding data are obtained from Beijing Nanjiao meteorological station during the same period in Beijing [9]. The smaller *R*_i_ means stronger shear force, indicating an unstable atmosphere that facilitates strong vertical mixing or turbulence, and vice versa.

Text 5: PMF

Positive matrix factorization (PMF) was employed to analyze the contributions of different sources to organic aerosol [10]. The PMF model is a bilinear model which can be defined as:

$X=GF+E$ (5)

where the measured matrix *X* is evaluated by the source component spectrum matrix (*F*), source contribution matrix (*G*), and the model residual (*E*) [10]. The PMF algorithm is solved in the weighted least squares sense, i.e., to minimize the weighted *E* which is divided by the matrix of the error of *X*. The PMF model requires all elements to be non-negative. Here the source finder (SoFi)[11] was used to solve the input data and analyze the results. The Unit Mass Resolution (UMR) matrix (m/z = 12-150) and error matrix were generated for organic species by using ToFware (Igor Pro; Wavemetrics, Inc.; Oregon, USA) [12]. The ions (m/z) were chosen up to 150 only for PMF analysis due to the lower signal-to-noise ratio (S/N) at larger m/z (>150).

PMF analysis was employed to deconvolve OA into six factors, including three primary OA components, cooking organic aerosol (COA), fossil fuel-related organic aerosol (FFOA), biomass burning organic aerosol (BBOA), and three secondary OA factors, less-oxidized oxygenated OA (LO-OOA), sulfate-related OOA (OOA-SO_4_^2-^) and nitrate-related OOA (OOA-NO_3_^-^). The mass spectra and times series of each OA factor are presented in Fig. S17 and Fig. S18.

FFOA was dominantly from coal combustion and traffic emissions. Since the mass spectra of coal combustion OA (CCOA) and hydrocarbon-like OA (HOA) are similar and difficult to separate, here we resolved FFOA as a mixed factor. The mass spectrum of FFOA is characterized by typical alkyl fragments (C_n_H_2n-1_^+^ and C_n_H_2n+1_^+^), e.g., 41, 43, 55, and 57. We also found a good correlation between FFOA and BC which is a primary emission tracer of combustion emissions (R^2^ = 0.61). FFOA accounts for 8.3% of OA, which is slightly lower than the 11.2% reported by Yao et al. [13], attributed to clean energy substitution in recent years.

The contribution of biomass burning to OA mass has been widely recognized [14, 15]. The BBOA factor accounts for 11.5% of OA in our study which is close to 13.0% reported by Yao et al. [13] and 11.7% reported by Ma et al. [2]. The mass spectrum of BBOA is characterized by the peaks at m/z 60, dominated by C_2_H_4_O_2_^+^, which is regarded as the fragment of levoglucosan and other related species [16]. The time series of BBOA has a good correlation with m/z 60 (R^2^ = 0.93).

The COA is a common OA component and has been widely reported [17-19]. The mass spectrum peaks at m/z 55 and 57 are the most remarkable characteristics of COA. We discovered that the time series of COA has a good correlation with m/z 55 (R^2^ = 0.65). COA showed an obvious diurnal pattern with two peaks, consistent with previous studies. The COA contributed 17.9% of total OA on average, which is comparable to previous studies [13]. Higher contribution of COA to total OA than FFOA and BBOA highlighting the importance of cooking emissions to OA.

The OOA-SO_4_^2-^ shows dominant mass spectrum peaks at m/z 29 and 31 which may be the organic fragments of hydroxymethanesulfonate (HMS, CH_2_(OH)SO_3_H)) [20] or organic sulfate. The OOA-SO_4_^2-^ was characterized by a good correlation with SO_4_^2-^ (R^2^ = 0.91), but the OOA-SO_4_^2-^ only contributed 3.9% of total OA, implying the minor contribution of the organic sulfate to OA.

The OOA-NO_3_^-^ shows a good correlation with NO_3_^-^ (R^2^ = 0.88), and characteristic peaks at m/z 28 and 44 which are typical of OOA. The O:C ratio of OOA-NO_3_^-^ is about 0.50. OOA-NO_3_^-^ accounts for 45.9% of the total mass of OA and is the largest factor in six OA factors.

The LOOA also has characteristic peaks at m/z 28 and 44, but has a low O:C ratio which is about 0.142. therefore we define it as LOOA. LOOA accounts for 12.5% of total OA.

Text 6: Sulfate Formation Rate Calculation

Secondary sulfate formation including multiphase oxidation by H_2_O_2_, O_3_, NO_2_, and TMI [21, 22], and heterogeneous reaction (gas-phase sulfuric acid (H_2_SO_4_) condensation) were calculated here.

Four multi-phase oxidation pathways of SO_2_ were considered, i.e., O_3_ oxidation, NO_2_ Oxidation, transition metal ions (TMIs) + O_2_ oxidation, and H_2_O_2_ oxidation. Considering the low contribution of methyl hydrogen peroxide oxidation and peroxyacetic acid oxidation [23], these two pathways were ignored. At the same time, due to some TMIs, such as Ti(Ⅲ), V(Ⅲ), Co(Ⅱ), Cu(Ⅱ) and Cr(Ⅲ), displayed much less catalytic activity [24], only Fe(Ⅲ) and Mn(Ⅱ) mere considered here. The sulfate formation rates were calculated as follows [24-26]:

$-\left( \frac{d[S\left( \mathrm{IV} \right)]}{dt} \right)_{O_{3}}=\left( k_{0}\left[ \mathrm{SO}_{2}H_{2}O \right]+k_{1}\left[ \mathrm{HSO}_{3}^{-} \right] \right.+k_{2}[\mathrm{SO}_{3}^{2-}])[O_{3(\mathrm{aq})}]$ (6)

${-\left( \frac{d\left[ S\left( \mathrm{IV} \right) \right]}{dt} \right)}_{H_{2}O_{2}}=\frac{k_{3}\left[ H^{+} \right]\left[ \mathrm{HSO}_{3}^{-} \right]\left[ H_{2}O_{2(\mathrm{aq})} \right]}{1+K\left[ H^{+} \right]}$ (7)

$-(\frac{d\left[ S\left( \mathrm{IV} \right) \right]}{dt})_{\mathrm{Fe}\left( \mathrm{IIII} \right)+\mathrm{Mn}\left( \mathrm{II} \right)}=k_{4}[H^{+}]^{a}[\mathrm{Mn}\left( \mathrm{II} \right)] \times[\mathrm{Fe}(\mathrm{III})][S(\mathrm{IV})]$ (8)

$-(\frac{d[S\left( \mathrm{IV} \right)]}{dt})_{\mathrm{NO}_{2}}=k_{5}[\mathrm{NO}_{2(\mathrm{aq})}][S\left( \mathrm{IV} \right)]$ (9)

where k_0_ = 2.4×10^4^ M^−1^ s^−1^, k_1_ = 3.7×10^5^ M^−1^ s^−1^, k_2_ = 1.5×10^9^ M^−1^ s^−1^, k_3_ = 7.45×10^7^ M^−1^ s^−1^, K = 13M^−1^, k_4_ = 3.72×10^7^ M^−1^ s^−1^, and a =−0.74 (for pH≤4.2) or k_4_ = 2.51×10^13^ M^−1^ s^−1^, and a = 0.67 (for pH > 4.2) and k_5_ = (1.24–1.67)×10^7^ M^−1^ s^−1^ (for 5.3 ≤ pH ≤ 8.7; the linear interpolated values were used for pH between 5.3 and 8.7) at 298K (Clifton et al., 1988); [O_3(aq)_], [H_2_O_2(aq)_] and [NO_2(aq)_] can be calculated by the Henry’s constants, which are 1.1×10^−2^, 1.0×10^5^ and 1.0×10^−2^ M atm^−1^ at 298K for O_3_, H_2_O_2_ and NO_2_ respectively (Seinfeld and Pandis, 2006). The detailed calculation method of Henry’s constants of HSO_3_^-^, SO_3_^2-^, and S(Ⅳ) can be found in the work reported by Liu et al. [26].

The sulfate formation rate from gas-phase sulfuric acid (H_2_SO_4_) condensation can be calculated as follows,

$J_{{SO}_{4}}=CS\times c_{H_{2}{SO}_{4}}$ (10)

$CS =2\pi D_{v}\int_{0}^{d_{p,max}} d_{p}\beta n\left( d_{p} \right)dd_{p}$ (11)

$\beta_{m}=\frac{1+{Kn}_{j}}{1+0.377{Kn}_{j}+1.33{Kn}_{j}(1+{Kn}_{j})/\alpha}$ (12)

where *J*_SO4_ is the sulfate formation rate from H_2_SO_4_ condensation, *CS* represents the first-order loss rate of H_2_SO_4_ onto particle surfaces, *c*_H2SO4_ is the concentration of H_2_SO_4_ vapor, *D_v_* represents the diffusion coefficient of H_2_SO_4_ vapor, *d*_p_ is the particle diameter; β is the dimensionless transitional correction factor for mass flux; *n*(d_p_) is the number concentration of particles of diameter *d*_p_. The concentration of H_2_SO_4_ was measured using a chemical ionization time-of-flight mass spectrometer equipped with a nitrate chemical ionization source (ToF-CIMS, Aerodyne Research, Inc. USA). Particle size from 3 nm to 10 μm were measured using a nano scanning mobility particle sizer (NSMPS, 3–60 nm), a regular scanning mobility particle sizer (RSMPS, 40–700 nm), and an aerodynamic particle sizer (APS, 550 nm–10 μm)[27]. The sulfuric acid concentration was calibrated once a month. The details of measurement and calibration can be found in previous studies [3, 28].

Text 7: OH concentration parameterization

The OH radical is a key parameter in atmospheric chemistry, as OH plays a central role in the oxidation of pollutants and trace gases. The production of OH in the troposphere is largely initiated by photolytic reactions, where *J*_O1D_ (the photolysis rate of O₃ producing O(¹D)) and *J*_NO2_ (the photolysis rate of NO₂) are critical. *J*_O1D_ determines the production rate of excited oxygen atoms (O(¹D)), which react with water vapor to form OH, while *J*_NO2_ governs the photolysis of NO₂, a prec ursor to O₃ and a contributor to secondary OH formation. Using *J*_O1D_ and *J*_NO2_ provides a direct and mechanistic approach to estimating OH concentrations, capturing the dynamic interaction between photochemistry and radical production under varying atmospheric conditions. A lot of research has used *J*_O1D_, *J*_NO2_, and NO_2_ concentration to estimate OH concentration [29-32], here we selected the method used by Zhang et al. [32], as follows:

$OH= \frac{4.1\times{10}^{9}\times\left( J_{{NO}_{2}} \right)^{0.19}\times\left( J_{O^{1}D} \right)^{0.83}\times\left( 140\left[ {NO}_{2} \right]+1 \right)}{0.41\left[ {NO}_{2} \right]^{2}+1.7\left[ {NO}_{2} \right]+1}$ （13）

Text 8: The details about the model settings

The WRF-Chem model (v4.0.3) was adopted to investigate the influence of ULOI events on the formation of sulfate. The simulation domain is the same of Zhao et al. [33] and centered at the BUCT station (Lat. 39º56ʹ31ʺ and Lon. 116º17ʹ52ʺ). The vertical resolution includes 30 layers with a fixed-model top pressure of 50 hPa, and the first layer was set to be about 17 m above the ground. The simulation period is from December 1, 2020, to February 28, 2021. The chemical mechanism used to simulate gases and aerosols is based on the Carbon Bond Mechanism version Z (CBMZ) scheme[34] coupled with the 4-bin sectional Model for Simulating Aerosol Interactions and Chemistry (MOSAIC) scheme[35]. The anthropogenic emissions are based on the multi-resolution emission inventory for China with base years of 2020 (MEIC), with a resolution of 0.25° × 0.25° (http://www.meicmodel.org/). The anthropogenic emissions from the MEIC have been processed to match the model grid spacing. Other detailed configurations can be found in Zhao et al[33].

**Reference**

1. Liu Y, Zhan J, Zheng F *et al.* Dust emission reduction enhanced gas-to-particle conversion of ammonia in the North China Plain. *Nature Communications*. 2022; **13**(1). doi: 10.1038/s41467-022-34733-4

2. Ma W, Zheng F, Zhang Y *et al.* Weakened Gas-to-Particle Partitioning of Oxygenated Organic Molecules in Liquified Aerosol Particles. *Environ Sci Technol Lett*. 2022; **9**(10): 837-843. doi: 10.1021/acs.estlett.2c00556

3. Liu Y, Yan C, Feng Z *et al.* Continuous and comprehensive atmospheric observations in Beijing: a station to understand the complex urban atmospheric environment. *Big Earth Data*. 2020; **4**(3): 295-321. doi: 10.1080/20964471.2020.1798707

4. Inness A, Ades M, Agustí-Panareda A *et al.* The CAMS reanalysis of atmospheric composition. *Atmos Chem Phys*. 2019; **19**(6): 3515-3556. doi: 10.5194/acp-19-3515-2019

5. Fountoukis C, Nenes A. ISORROPIA II: a computationally efficient thermodynamic equilibrium model for K^+^-Ca^2+^-Mg^2+^-NH_4_^+^-Na^+^-SO_4_^2-^-NO_3_^-^-Cl^-^-H_2_O aerosols. *Atmos Chem Phys*. 2007; **7**(17): 4639-4659. doi: 10.5194/acp-7-4639-2007

6. Masiol M, Squizzato S, Formenton G *et al.* Hybrid multiple-site mass closure and source apportionment of PM_2.5_ and aerosol acidity at major cities in the Po Valley. *Sci Total Environ*. 2020; **704**: 135287. doi: <https://doi.org/10.1016/j.scitotenv.2019.135287>

7. Vogelezang DHP, Holtslag AAM. Evaluation and model impacts of alternative boundary-layer height formulations. *Boundary-Layer Meteorology*. 1996; **81**(3): 245-269. doi: 10.1007/BF02430331

8. Sicard M, Pérez C, Rocadenbosch F *et al.* Mixed-Layer Depth Determination in the Barcelona Coastal Area From Regular Lidar Measurements: Methods, Results and Limitations. *Boundary-Layer Meteorology*. 2006; **119**(1): 135-157. doi: 10.1007/s10546-005-9005-9

9. Liao Z, Pan Y, Ma P *et al.* Meteorological and chemical controls on surface ozone diurnal variability in Beijing: A clustering-based perspective. *Atmos Environ*. 2023; **295**: 119566. doi: <https://doi.org/10.1016/j.atmosenv.2022.119566>

10. Paatero P, Tapper U. Positive matrix factorization: A non-negative factor model with optimal utilization of error estimates of data values. *Environmetrics*. 1994; **5**(2): 111-126. doi: <https://doi.org/10.1002/env.3170050203>

11. Canonaco F, Crippa M, Slowik JG *et al.* SoFi, an IGOR-based interface for the efficient use of the generalized multilinear engine (ME-2) for the source apportionment: ME-2 application to aerosol mass spectrometer data. *Atmos Meas Tech*. 2013; **6**(12): 3649-3661. doi: 10.5194/amt-6-3649-2013

12. Ulbrich IM, Canagaratna MR, Zhang Q *et al.* Interpretation of organic components from Positive Matrix Factorization of aerosol mass spectrometric data. *Atmos Chem Phys*. 2009; **9**(9): 2891-2918. doi: 10.5194/acp-9-2891-2009

13. Xiao Y, Hu M, Zong T *et al.* Insights into aqueous-phase and photochemical formation of secondary organic aerosol in the winter of Beijing. *Atmos Environ*. 2021; **259**: 118535. doi: <https://doi.org/10.1016/j.atmosenv.2021.118535>

14. Wang J, Ye J, Zhang Q *et al.* Aqueous production of secondary organic aerosol from fossil-fuel emissions in winter Beijing haze. *Proc Natl Acad Sci USA*. 2021; **118**(8): e2022179118. doi: doi:10.1073/pnas.2022179118

15. Lei L, Zhou W, Chen C *et al.* Long-term characterization of aerosol chemistry in cold season from 2013 to 2020 in Beijing, China. *Environ Pollut*. 2021; **268**: 115952. doi: <https://doi.org/10.1016/j.envpol.2020.115952>

16. Alfarra MR, Prevot ASH, Szidat S *et al.* Identification of the Mass Spectral Signature of Organic Aerosols from Wood Burning Emissions. *Environ Sci Technol*. 2007; **41**(16): 5770-5777. doi: 10.1021/es062289b

17. Li YJ, Sun Y, Zhang Q *et al.* Real-time chemical characterization of atmospheric particulate matter in China: A review. *Atmos Environ*. 2017; **158**: 270-304. doi: 10.1016/j.atmosenv.2017.02.027

18. Huang RJ, Wang Y, Cao J *et al.* Primary emissions versus secondary formation of fine particulate matter in the most polluted city (Shijiazhuang) in North China. *Atmos Chem Phys*. 2019; **19**(4): 2283-2298. doi: 10.5194/acp-19-2283-2019

19. Li X, Wu J, Elser M *et al.* Contributions of residential coal combustion to the air quality in Beijing–Tianjin–Hebei (BTH), China: a case study. *Atmos Chem Phys*. 2018; **18**(14): 10675-10691. doi: 10.5194/acp-18-10675-2018

20. Dovrou E, Lim CY, Canagaratna MR *et al.* Measurement techniques for identifying and quantifying hydroxymethanesulfonate (HMS) in an aqueous matrix and particulate matter using aerosol mass spectrometry and ion chromatography. *Atmos Meas Tech*. 2019; **12**(10): 5303-5315. doi: 10.5194/amt-12-5303-2019

21. Liu T, Chan AWH, Abbatt JPD. Multiphase Oxidation of Sulfur Dioxide in Aerosol Particles: Implications for Sulfate Formation in Polluted Environments. *Environ Sci Technol*. 2021; **55**(8): 4227-4242. doi: 10.1021/acs.est.0c06496

22. Peng J, Hu M, Shang D *et al.* Explosive Secondary Aerosol Formation during Severe Haze in the North China Plain. *Environ Sci Technol*. 2021; **55**(4): 2189-2207. doi: 10.1021/acs.est.0c07204

23. Zheng B, Zhang Q, Zhang Y *et al.* Heterogeneous chemistry: a mechanism missing in current models to explain secondary inorganic aerosol formation during the January 2013 haze episode in North China. *Atmos Chem Phys*. 2015; **15**(4): 2031-2049. doi: 10.5194/acp-15-2031-2015

24. Cheng Y, Zheng G, Wei C *et al.* Reactive nitrogen chemistry in aerosol water as a source of sulfate during haze events in China. *Science Advances*. 2016; **2**(12): e1601530. doi: doi:10.1126/sciadv.1601530

25. Liu Y, Feng Z, Zheng F *et al.* Ammonium nitrate promotes sulfate formation through uptake kinetic regime. *Atmos Chem Phys*. 2021; **21**(17): 13269-13286. doi: 10.5194/acp-21-13269-2021

26. Liu P, Ye C, Xue C *et al.* Formation mechanisms of atmospheric nitrate and sulfate during the winter haze pollution periods in Beijing: gas-phase, heterogeneous and aqueous-phase chemistry. *Atmos Chem Phys*. 2020; **20**(7): 4153-4165. doi: 10.5194/acp-20-4153-2020

27. Liu J, Jiang J, Zhang Q *et al.* A spectrometer for measuring particle size distributions in the range of 3 nm to 10 μm. *Front Environ Sci Eng*. 2016; **10**(1): 63-72. doi: 10.1007/s11783-014-0754-x

28. Wang Y, Ma Y, Yan C *et al.* Sulfur Dioxide Transported From the Residual Layer Drives Atmospheric Nucleation During Haze Periods in Beijing. *Geophys Res Lett*. 2023; **50**(6): e2022GL100514. doi: <https://doi.org/10.1029/2022GL100514>

29. Ehhalt DH, Rohrer F. Dependence of the OH concentration on solar UV. *J Geophys Res*. 2000; **105**(D3): 3565-3571. doi: <https://doi.org/10.1029/1999JD901070>

30. Alicke B, Platt U, Stutz J. Impact of nitrous acid photolysis on the total hydroxyl radical budget during the Limitation of Oxidant Production/Pianura Padana Produzione di Ozono study in Milan. *J Geophys Res*. 2002; **107**(D22): LOP 9-1-LOP 9-17. doi: <https://doi.org/10.1029/2000JD000075>

31. Liu Y, Zhang Y, Lian C *et al.* The promotion effect of nitrous acid on aerosol formation in wintertime in Beijing: the possible contribution of traffic-related emissions. *Atmos Chem Phys*. 2020; **20**(21): 13023-13040. doi: 10.5194/acp-20-13023-2020

32. Zhang Y, Zheng F, Feng Z *et al.* Concentration and source changes of nitrous acid (HONO) during the COVID-19 lockdown in Beijing. *Atmos Chem Phys*. 2024; **24**(15): 8569-8587. doi: 10.5194/acp-24-8569-2024

33. Zhao X, Zhao X, Chen D *et al.* Seasonal simulation and source apportionment of SO42− with integration of major highlighted chemical pathways in WRF-Chem model in the NCP. *Atmospheric Pollution Research*. 2024; **15**(11): 102268. doi: <https://doi.org/10.1016/j.apr.2024.102268>

34. Zaveri RA, Peters LK. A new lumped structure photochemical mechanism for large-scale applications. *J Geophys Res*. 1999; **104**(D23): 30387-30415. doi: <https://doi.org/10.1029/1999JD900876>

35. Zaveri RA, Easter RC, Fast JD *et al.* Model for Simulating Aerosol Interactions and Chemistry (MOSAIC). *J Geophys Res*. 2008; **113**(D13). doi: <https://doi.org/10.1029/2007JD008782>

Table S1.

Summary of variation of meteorological factors, gas pollutants, and particulate matter during the study period at BUCT station

| Parameters | Median± Standard deviation | | | |
| --- | --- | --- | --- | --- |
|  | Non ULOIs | Weak ULOIs | Moderate ULOIs | Strong ULOIs |
| T (℃) | 3.53±5.15 | 4.91±8.25 | 7.12±7.16 | 6.91±7.47 |
| RH (%) | 42.82±21.73 | 41.29±24.19 | 40.14±23.72 | 33.16±19.76 |
| UVA (W/m^2^) | 4.21±6.80 | 4.74±7.64 | 4.58±7.41 | 5.86±9.05 |
| UVB (W/m^2^) | 0.042±0.078 | 0.053±0.10 | 0.046±0.084 | 0.058±0.11 |
| Pressure (Pa) | 1013.84±6.48 | 1016.05±7.47 | 1012.02±7.55 | 1010.19±6.26 |
| WS (m/s) | 0.80±0.55 | 0.93±0.81 | 0.85±0.66 | 0.88±0.67 |
| O_3_ (ppbv) | 5.65±8.16 | 11.24±10.94 | 17.50±9.51 | 22.70±7.73 |
| NO (ppbv) | 27.19±28.04 | 9.18±14.26 | 2.56±4.80 | 1.07±3.20 |
| NO_2_ (ppbv) | 30.45±12.20 | 25.08±13.43 | 15.71±11.81 | 9.02±8.89 |
| NOx (ppbv) | 57.57±36.25 | 34.90±22.99 | 17.77±14.78 | 19.00±10.73 |
| CO (ppbv) | 1151.63±433.38 | 877.92±415.44 | 671.05±448.04 | 515.32±284.42 |
| SO_2_ (ppbv) | 2.13±1.51 | 2.04±1.14 | 1.65±1.18 | 1.34±1.07 |
| PM_10_ (μg/m^3^) | 64.87±25.21 | 54.30±26.90 | 48.26±39.77 | 38.16±39.92 |
| PM_2.5_ (μg/m^3^) | 48.13±23.71 | 46.99±26.61 | 39.70±40.24 | 26.85±32.97 |
| SO_4_^2-^ (μg/m^3^) | 3.58±3.99 | 3.25±4.51 | 3.50±5.53 | 1.64±2.47 |
| NO_3_- (μg/m^3^) | 12.23±10.73 | 9.60±8.92 | 7.75±10.46 | 3.12±6.55 |
| NH_4_^+^ (μg/m^3^) | 5.00±4.17 | 4.21±4.17 | 3.69±5.09 | 1.59±2.87 |
| Cl^-^ (μg/m^3^) | 0.87±0.67 | 0.71±0.62 | 0.75±1.25 | 0.35±0.85 |
| OA (μg/m^3^) | 9.59±5.45 | 7.67±4.66 | 6.68±7.27 | 4.00±5.17 |

Table S2.

Instruments used in the BUCT station

| Parameters | Instruments |
| --- | --- |
| PM_2.5_ mass | Taper Element Oscillating Microbalance (TEOM, 1405-DF, Thermo Fisher) |
| Water-soluble ions | Monitoring AeRosols and Gases in Ambient Air (MARGA 2060R, Metronhm Process Analytics) |
| Non-refractory PM_2.5_ composition | Time-of-Flight Aerosol Chemical Speciation Monitor (ToF-ACSM, Aerodyne) |
| Weather conditions | Weather station (AWS310, Vaisala) |
| Trace gases | NOx, SO_2_, CO and O_3_ analyzer (42i, 43i, 48i and 49i, Thermo Scientific) |


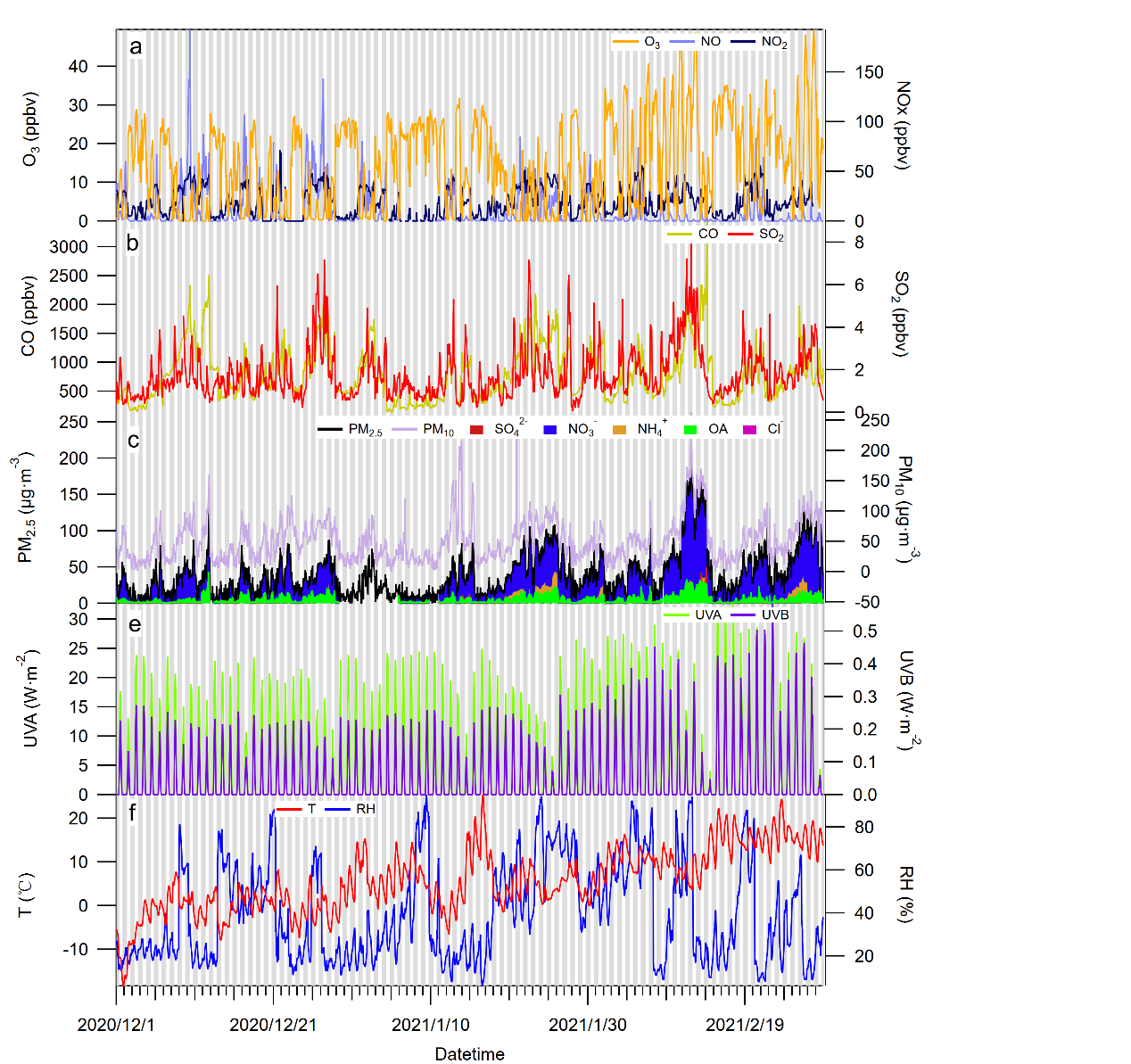


Fig. S1.

The time series of gas pollutants and particulate matter during observation at BUCT station.


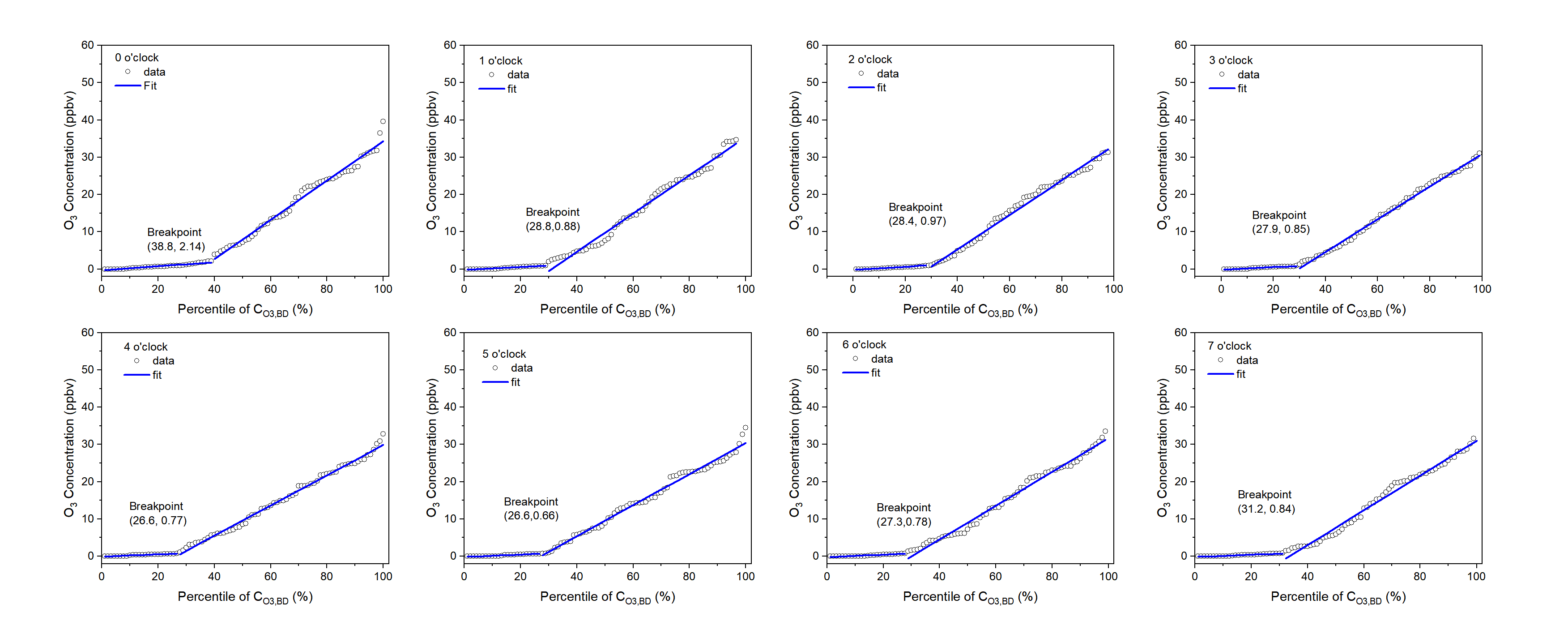


Fig. S2.

The scatter plot of O_3_ concentration against its percentiles and the fitting result of ULOI and NULOI from 0 o’clock to 7 o’clock.





Fig. S3.

Scatter plot of O_3_ concentration as a function of O_3_ Ranking for 4:00-6:00 am at other observation station


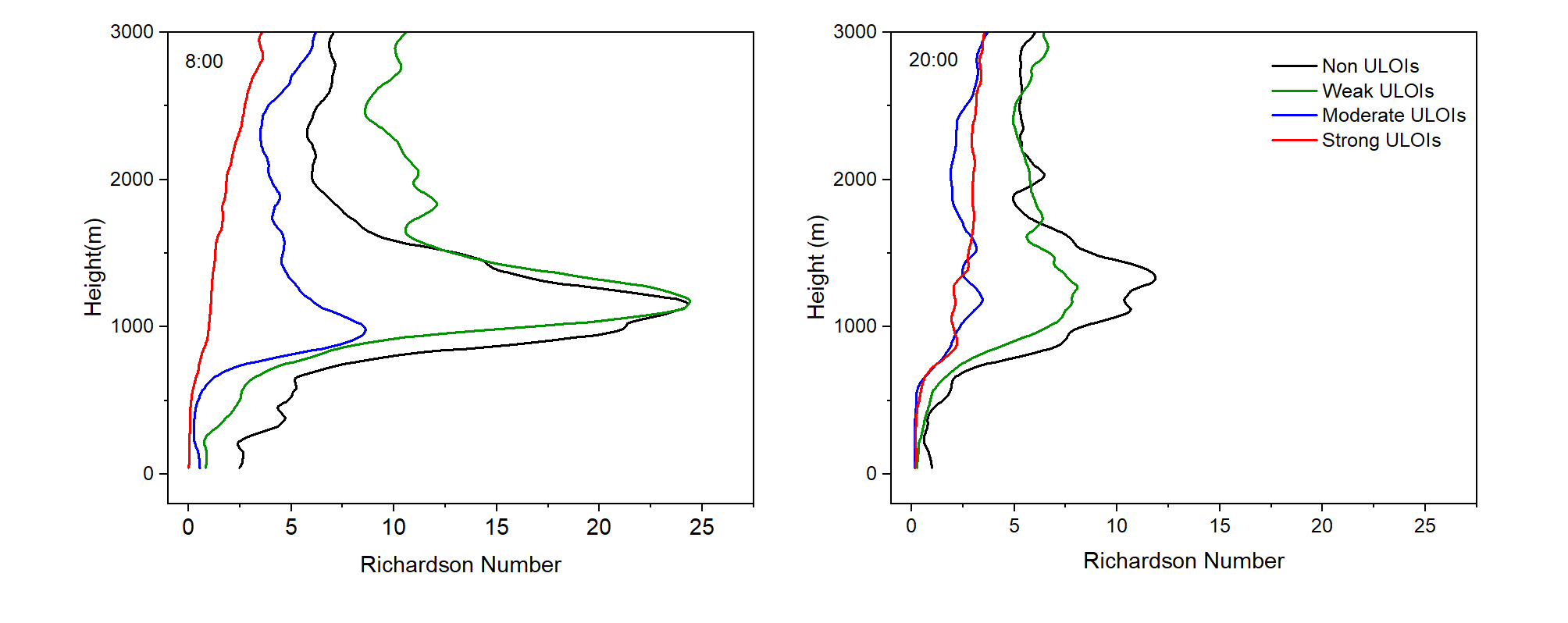


Fig. S4.

The smoothed vertical profile of the Richardson number at 8:00 and the previous day’s 20:00 (local time) under different intensities of ULOIs.


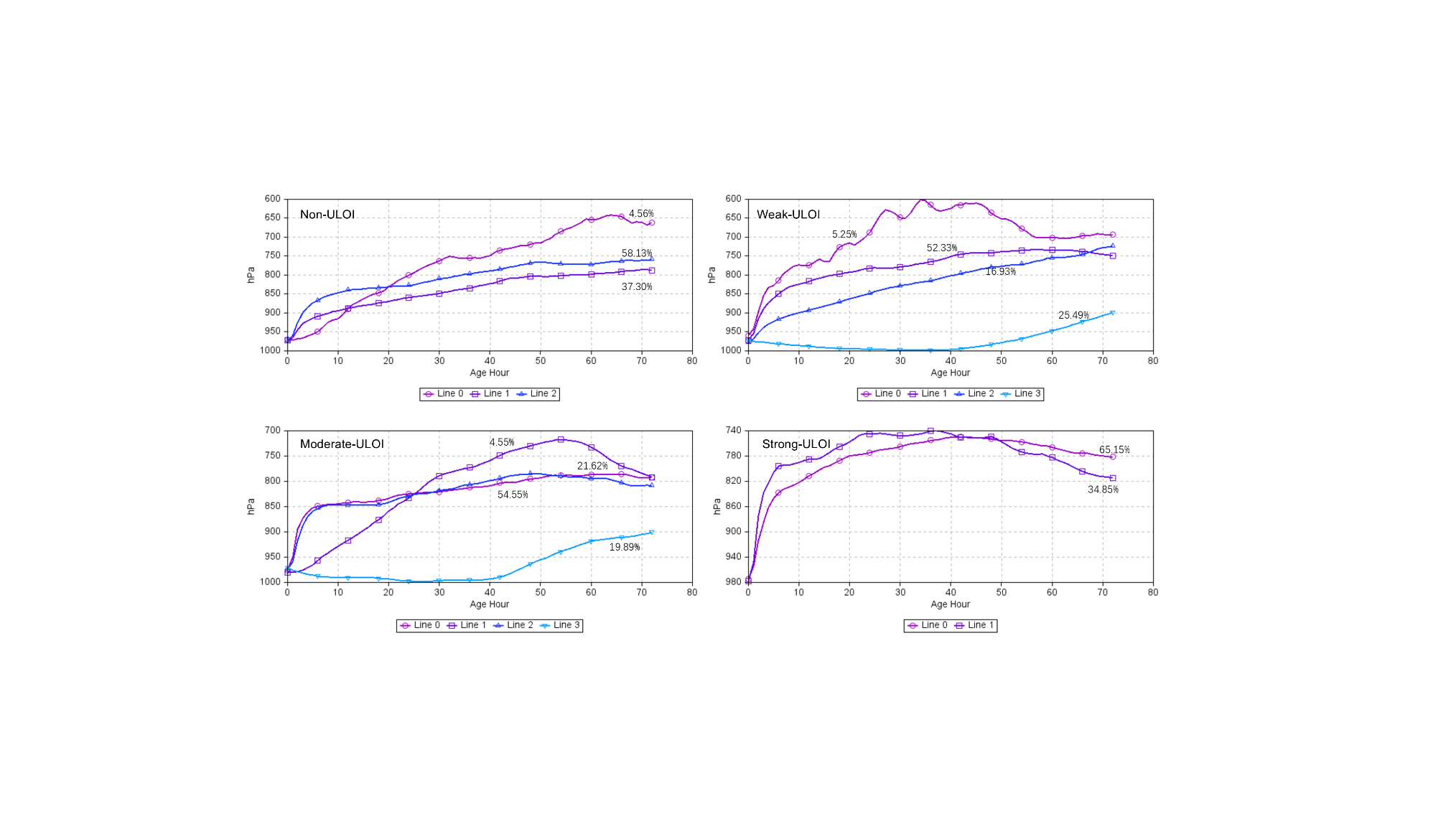


Fig. S5.

Height variations of 72-hour backward trajectories during different ULOI events. The number represents the fraction of airmass during the events.


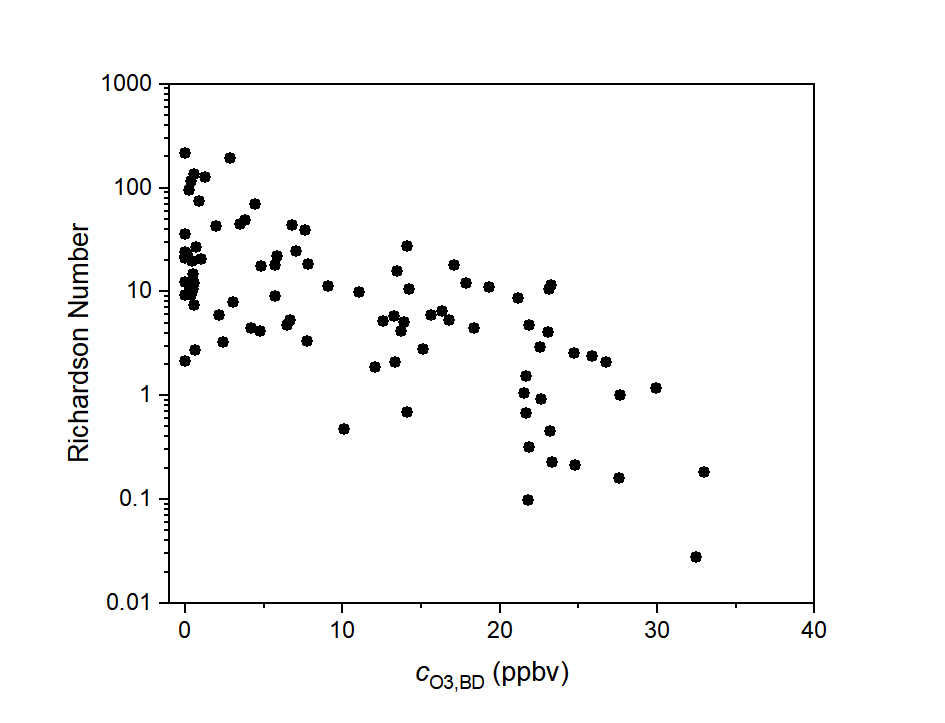


Fig. S6.

The distribution of peaks of Richardson numbers between 700 and 1500 m as a function of *c*_O3,BD_.





Fig. S7.

Vertical distribution of O_3_ flux at different intensities of ULOI. (a) Non-ULOIs, (b) weak ULOIs, (c) moderate ULOIs, (d) strong ULOIs.


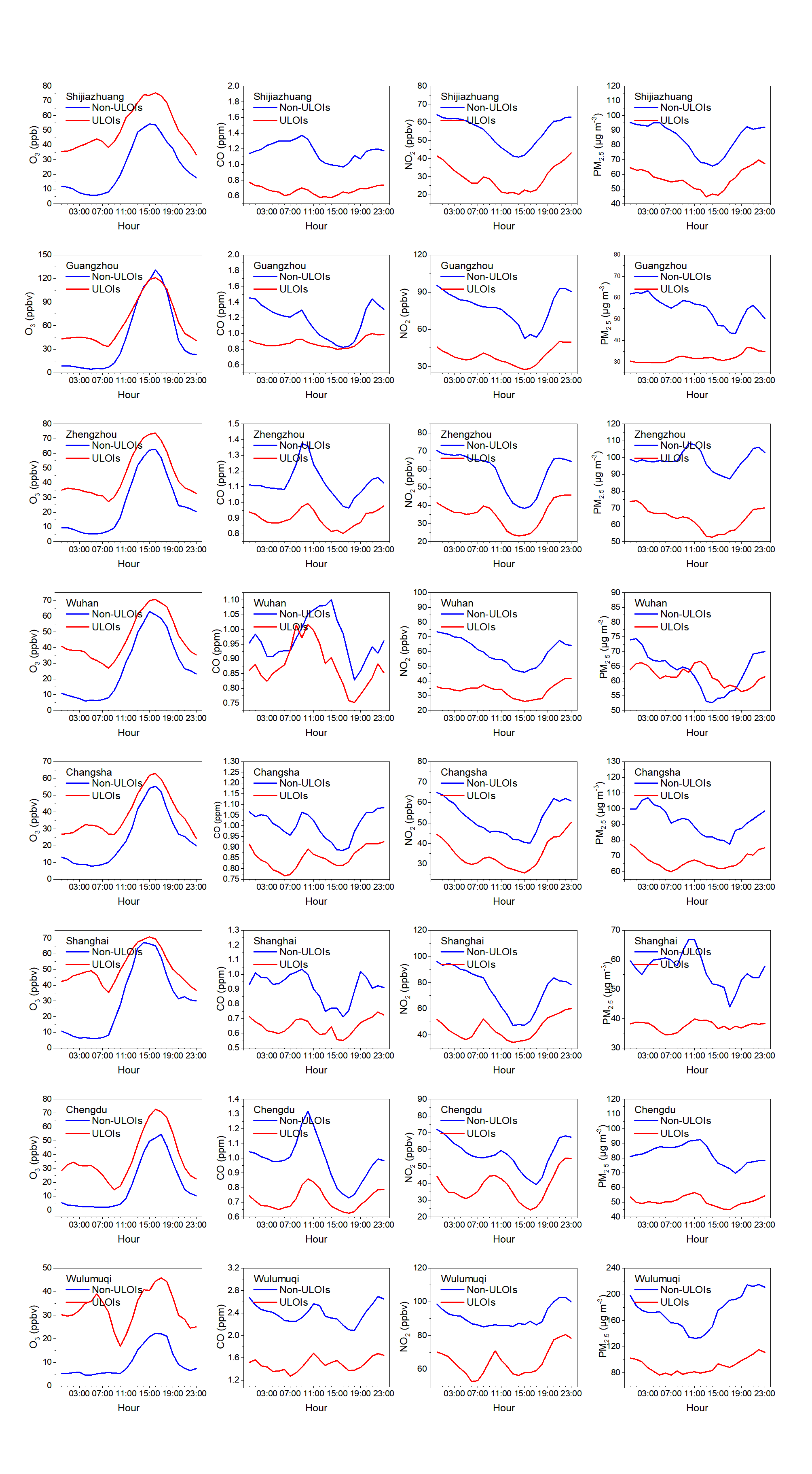


Fig. S8.

The diurnal variation of O_3_, CO, NO_2_, and PM_2.5_ during non-ULOIs and ULOIs at typical stations (Shijiazhuang, Guangzhou, Zhengzhou, Wuhan, Changsha, Shanghai, Chengdu, and Wulumuqi) from December 2020 to February 2021.





Fig. S9.

The mass fraction of NR-PM_2.5_ species in different ULOI intensity.


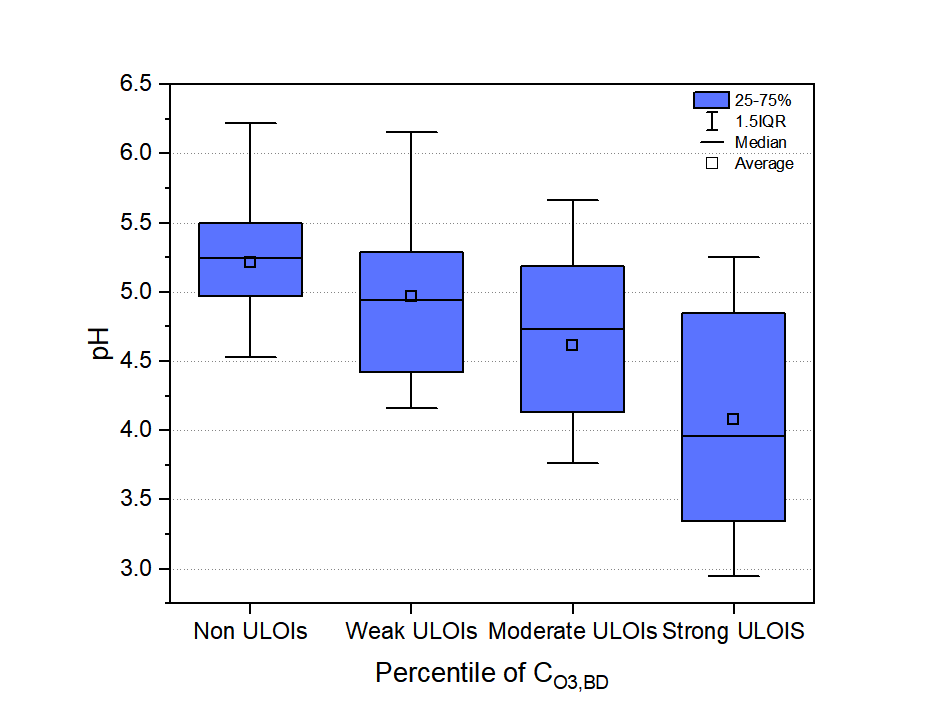


Fig. S10.

Aerosol pH in different ULOI intensifies.


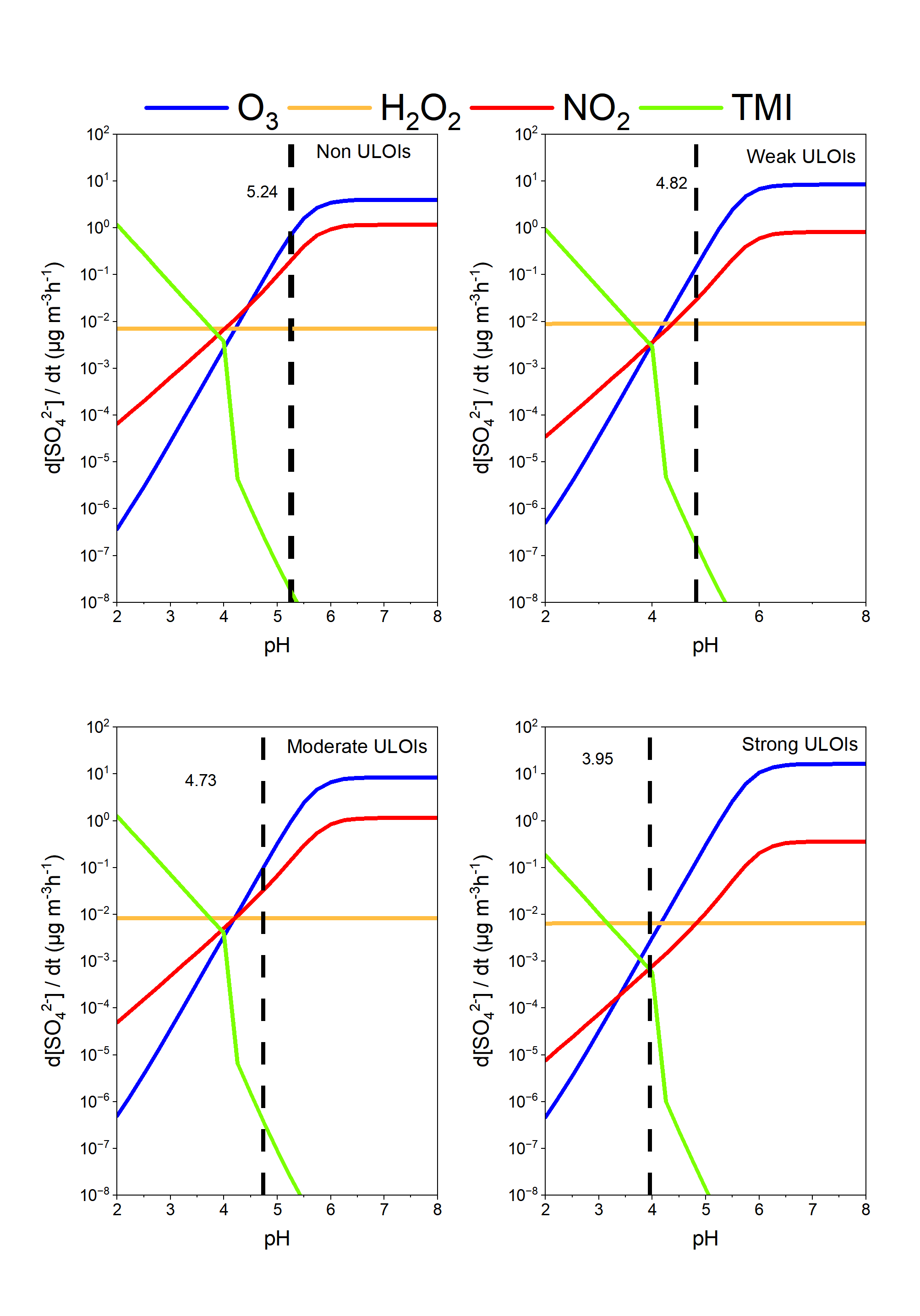


Fig. S11.

The sensitivity of sulfate production to aerosol pH under different intensities of ULOIs in Beijing. (a) Non ULOIs, (b) Weak ULOIs, (c)Moderate ULOIs, and (d) Strong ULOIs. The dashed line represents the median pH.


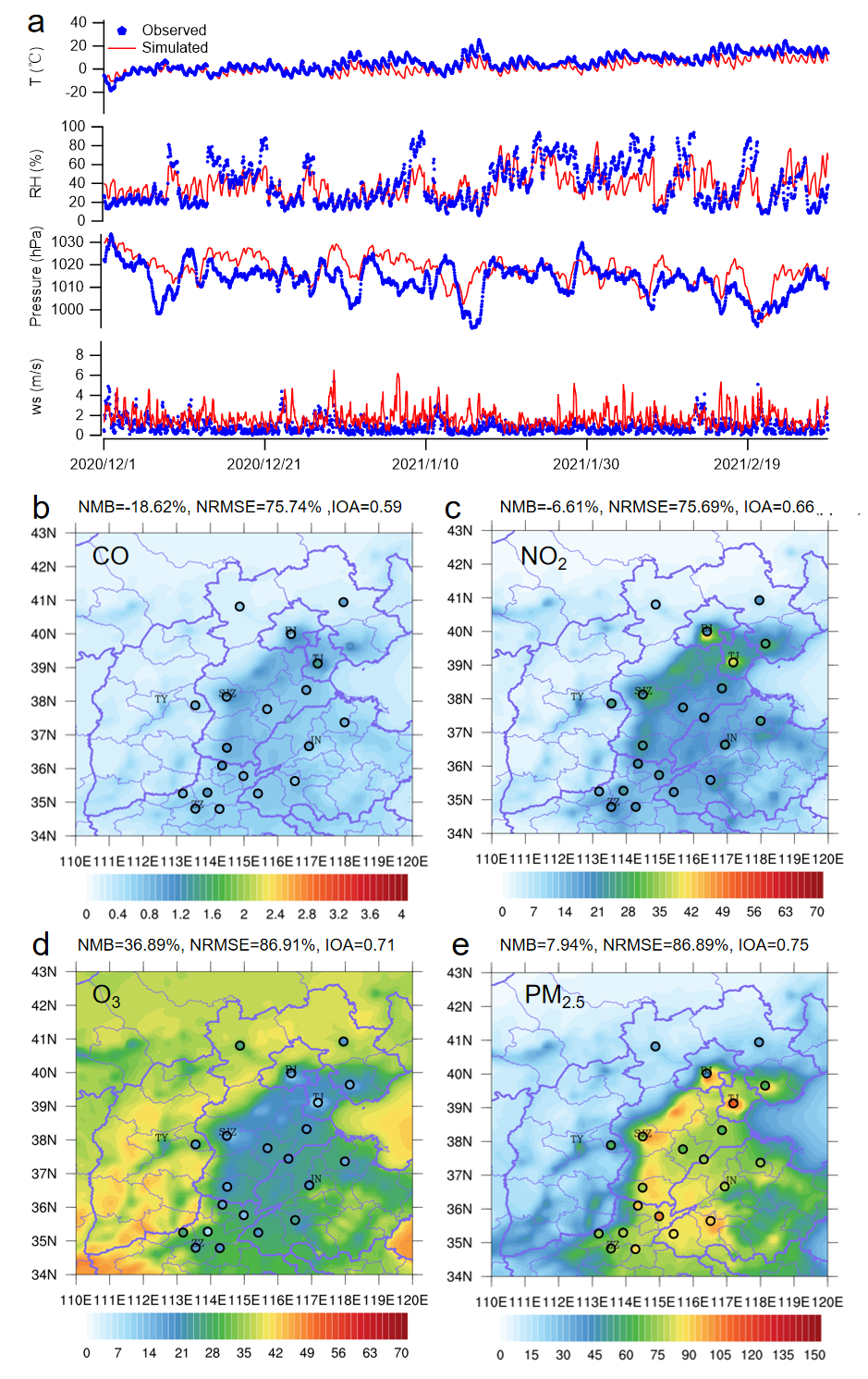


Fig. S12.

(a) The time series of observed and simulated meteorological parameters (temperature, relative humidity, pressure, and wind speed) at the BUCT station. (b-e) Contours of simulated concentrations of CO, NO2, O3, and PM2.5 against the observation data from national air quality monitoring sites.


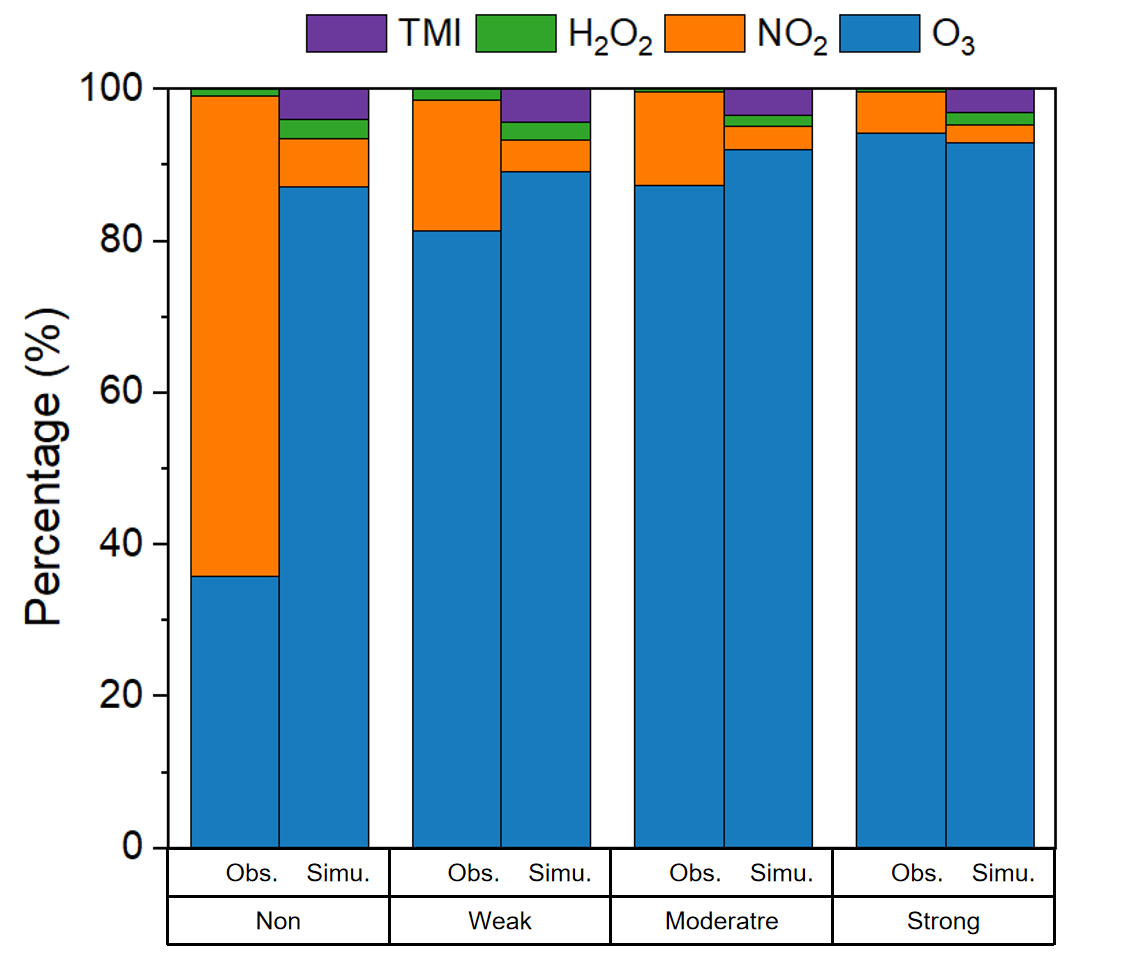


Fig. S13.

The percentage of sulfate formation rates via different formation pathways in a liquid aerosol.


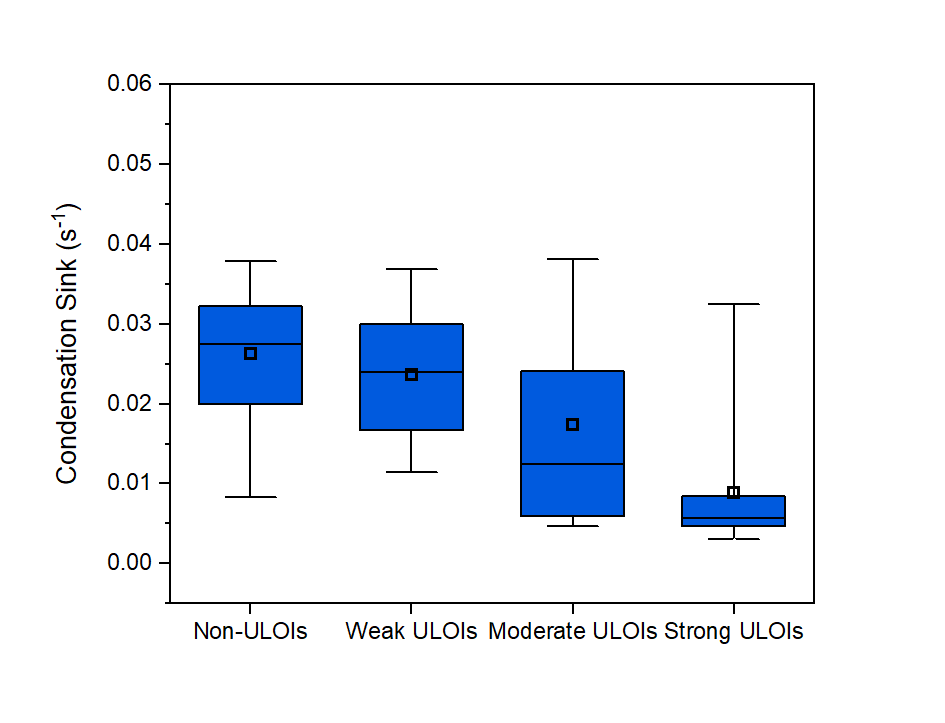


Fig. S14.

The dependence of the condensation sink (CS) on the different intensities of the ULOI at the BUCT station.


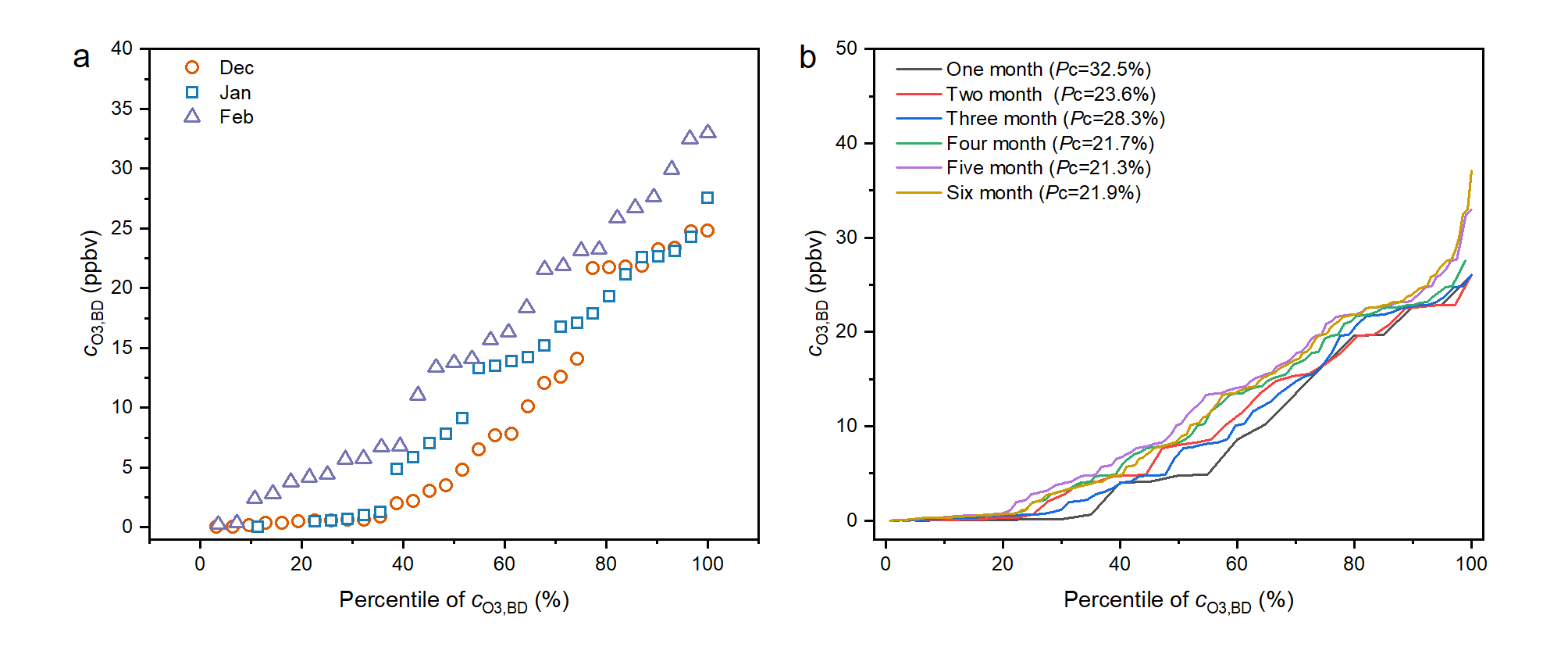


Fig. S15.

The sensitivity of Pc to the length of the dataset. a, *c*_O3,BD_ and their percentile distribution for December, January, or February, shown separately for each month. b, *c*_O3,BD_ and their percentile distribution six months combined (from October to March of the following year).


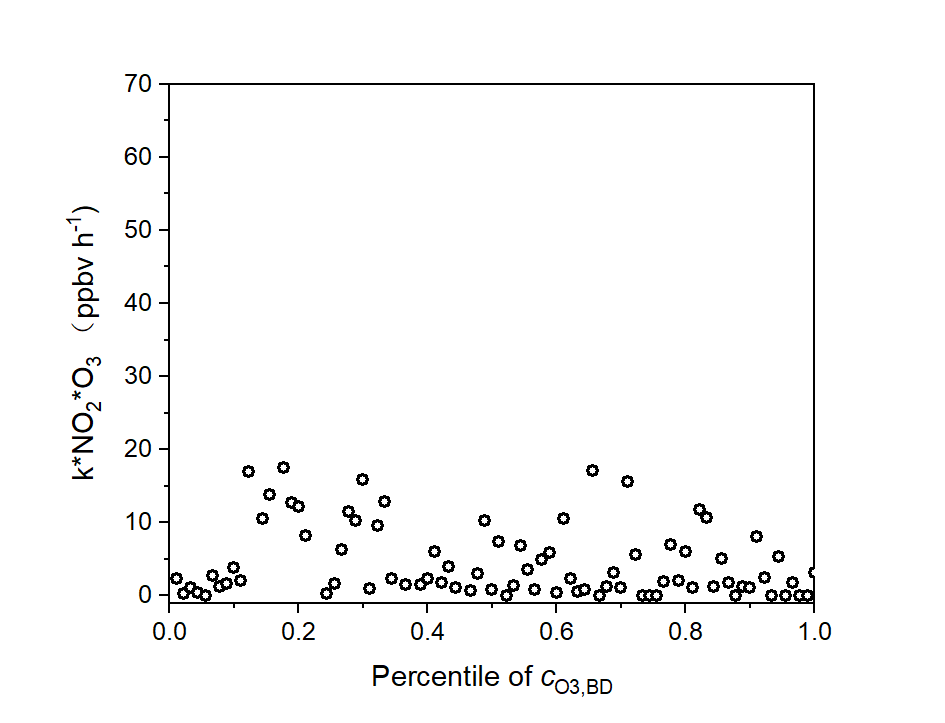


Fig. S16.

The scatter plot of nighttime titration rate of O_3_ against the percentiles of *c*_O3,BD_ at BUCT station.


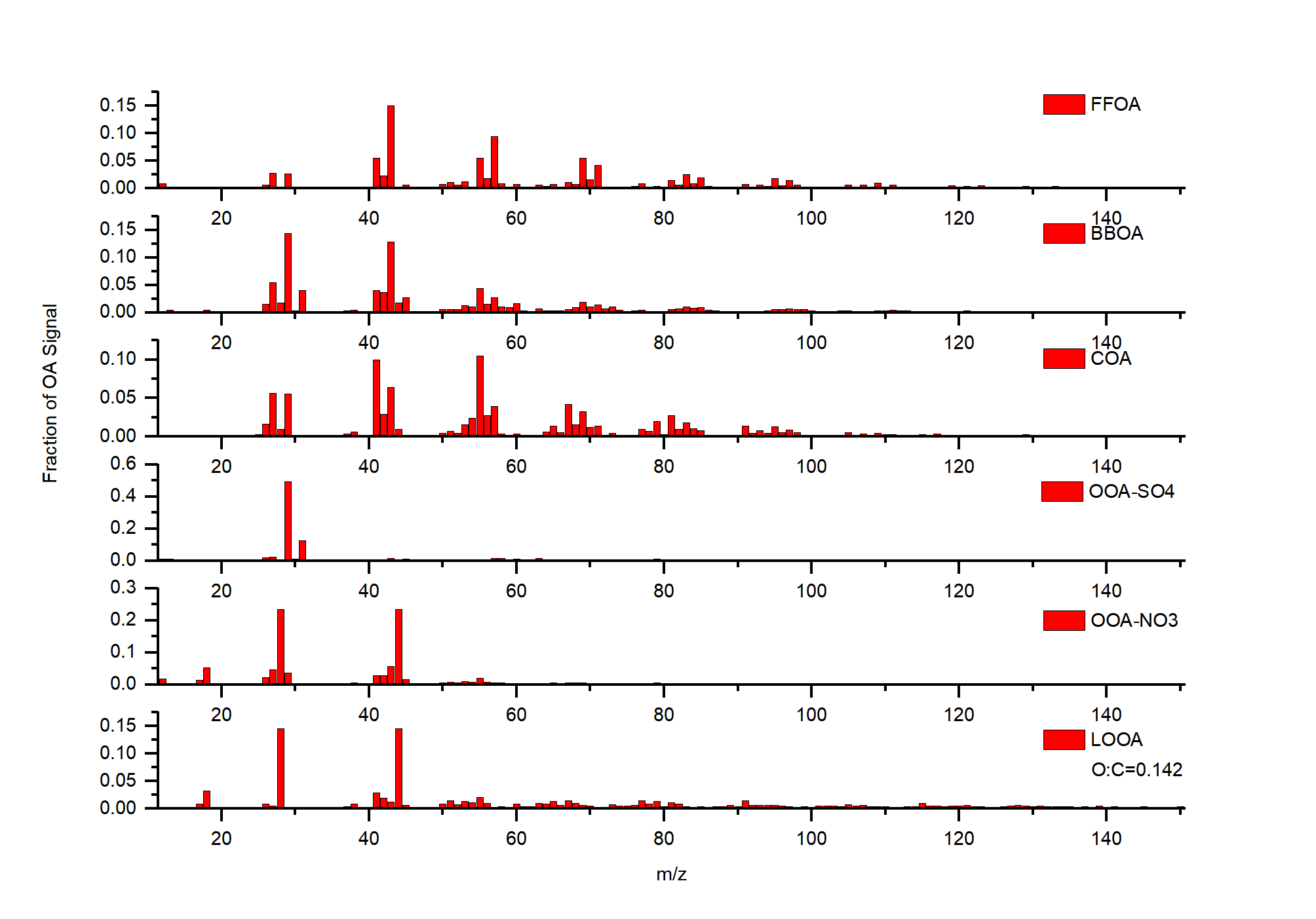


Fig. S17.

The characteristics of mass spectra for each OA factor by PMF.


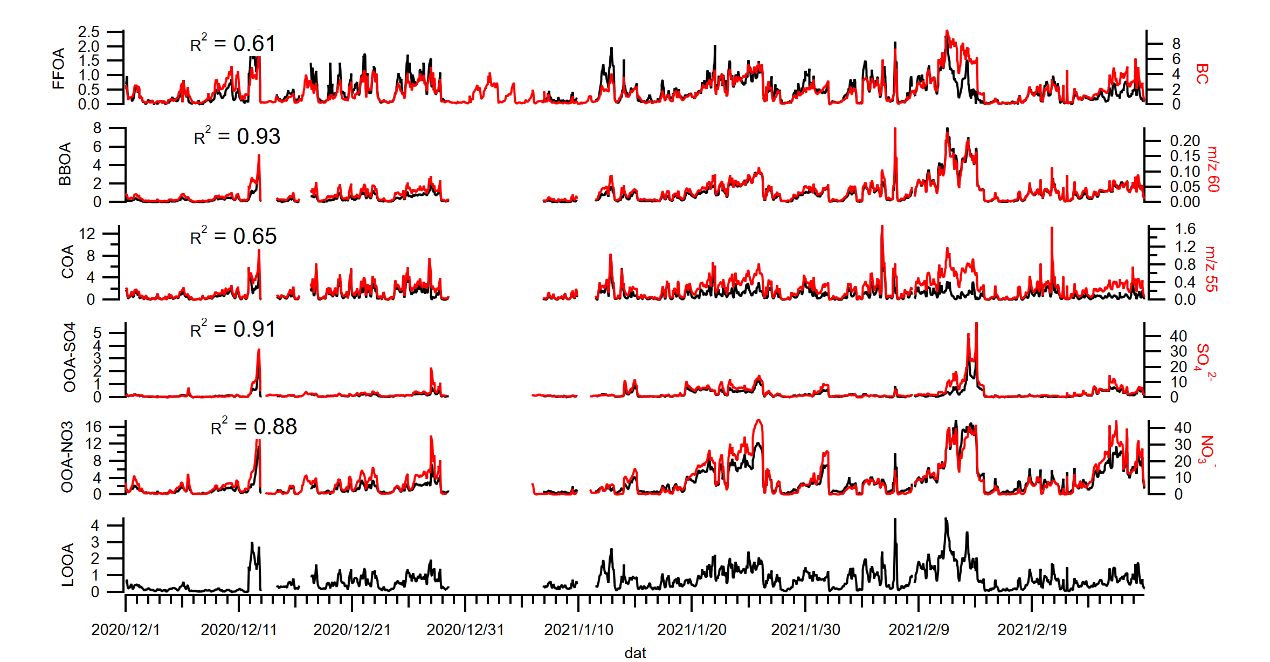


Fig. S18.

The time series of six OA factors and other relevant species at BUCT station. Pearson correlation coefficients squared (R^2^) between each OA factor and corresponding species are also added.
